# A First Draft of the Core Fungal Microbiome of *Schedonorus arundinaceus* with and without Its Fungal Mutualist *Epichloë coenophiala*

**DOI:** 10.3390/jof8101026

**Published:** 2022-09-28

**Authors:** Jenna C. M. Dale, Jonathan A. Newman

**Affiliations:** 1 Department of Integrative Biology, University of Guelph, Guelph, ON N1G 2W1, Canada; 2 Department of Biology, Wilfrid Laurier University, Waterloo, ON N2L 3C5, Canada

**Keywords:** *Schedonorus arundinaceus*, *Epichloë coenophiala*, microbiome, endophyte, phyllosphere, foliar fungi

## Abstract

Tall fescue (*Schedonorus arundinaceus*) is a cool-season grass which is commonly infected with the fungal endophyte *Epichloë coenophiala*. Although the relationship between tall fescue and *E. coenophiala* is well-studied, less is known about its broader fungal communities. We used next-generation sequencing of the ITS2 region to describe the complete foliar fungal microbiomes in a set of field-grown tall fescue plants over two years, and whether these fungal communities were affected by the presence of *Epichloë*. We used the Georgia 5 cultivar of tall fescue, grown in the field for six years prior to sampling. Plants were either uninfected with *E. coenophiala*, or they were infected with one of two *E. coenophiala* strains: The common toxic strain or the AR542 strain (sold commerically as MaxQ). We observed 3487 amplicon sequence variants (ASVs) across all plants and identified 43 ASVs which may make up a potential core microbiome. Fungal communities did not differ strongly between *Epichloë* treatments, but did show a great deal of variation between the two years. Plant fitness also changed over time but was not influenced by *E. coenophiala* infection.

## 1. Introduction

Plants are home to a wide variety of microorganisms which impact their survival and function. Research into the plant microbiome indicates that plants often contain hundreds to thousands of fungal and bacterial species, ranging from beneficial (mutualistic) to harmful (parasitic).

Tall fescue (*Schedonorus arundinaceus* (Schreb.) Dumort., formerly *Festuca arundinacea* Schreb., *Lolium arundinaceum* (Schreb.) Darbysh., *Schedonorus phoenix* (Scop.) Holub) is a cool-season grass native to Europe and northern Africa, and is widely cultivated throughout the temperate world for use as a forage and turf grass [1]. Tall fescue is commonly infected with the fungal endophyte *Epichloë coenophiala* (Morgan-Jones & W. Gams) C.W. Bacon & Schardl (formerly *Neotyphodium coenophialum* (Morgan-Jones & W. Gams) Glenn, C.W. Bacon & Hanlin [2]). This endophyte is asexual and strictly vertically transmitted, meaning that it can only be transmitted from mother to daughter via the seed. As a result of this vertical transmission, the relationship between tall fescue and *E. coenophiala* is mutualistic, although depending on the environmental conditions the benefits may not always outweigh the costs [3].

Benefits of *E. coenophiala* are attributed to its production of alkaloids (although other factors may also contribute [4,5,6]), and include resistance to insect and mammalian herbivory, drought, and some plant pathogens [7,8,9]. Resistance to mammalian herbivory in particular is caused by ergot alkaloids, which make grass containing the common toxic strain of *E. coenophiala* less desirable for use with livestock. This has led to the development of cultivars containing so-called “novel” endophytes (natural, but uncommon strains of *E. coenophiala*). Some of these novel endophytes do not produce the ergot alkaloids, but still confer some of the other benefits which improve plant growth and survival [10,11].

Beyond *Epichloë*, research into associations between fungi and tall fescue has typically focused on fungal pathogens [9,12,13,14,15,16], soil fungi [17,18,19], and root fungi such as arbuscular mycorrhizal fungi (AMF) and dark septate endophytes [20,21,22,23,24,25]. Many (although not all, see [16,22,24,25]) of these studies also show correlations between *Epichloë* infection and the fungus being studied.

Fewer studies focus on the aboveground (or foliar) portion of the tall fescue fungal microbiome. Nissinen et al. [26] observed 54 fungal OTUs (operational taxonomic units) in tall fescue leaves and found that these communities were influenced by *E. coenophiala* infection. In the closely related grass and fungal species perennial ryegrass (*Lolium perenne* L.) and *Epichloë festucae* var. *lolii* C.W. Bacon & Schardl (formerly *Neotyphodium lolii* (M.J. Latch Chr. & Samuels) Glenn, C.W. Bacon & Hanlin, *Acremonium lolii* M.J. Latch Chr. & Samuels), König et al. [27] observed 247 OTUs but did not observe an effect of the *Epichloë* endophyte on fungal community composition. The communities were, however, strongly affected by study region and season. Liu et al. [28] observed 479 OTUs in drunken horse grass (*Achnatherum inebrians* (Hance) Keng) and found no effect of *Epichloë gansuensis* (C.J. Li & Na) Schardl (formerly *Neotyphodium gansuense* C.J. Li & Nan) on fungal communities, but did observe a difference between endophytic and epiphytic communities. From these few studies, it appears that cool-season grasses, like many other plants, contain diverse fungal microbiomes. It is not yet clear how important these communities are to their hosts, but research into other plant species has shown effects of foliar fungi on factors such as seed production, disease severity, and nitrogen uptake [29,30,31].

In this study, we sought to extend the work of Nissinen et al. [26] by examining tall fescue plants that had been established in the field for much longer (6 years vs. 3 months) and following individual plants for multiple years. We used next-generation sequencing of the fungal ITS2 region, and asked and answered the following questions.

1. What species are present?
2. What species were present in all or most plants over time (which we refer to as the draft “core microbiome”)?
3. Were these fungal communities influenced by the presence of the *E. coenophiala* endophyte?
4. Were these fungal communities different between plants infected with different strains of the *Epichloë* endophyte?
5. Were these fungal communities correlated with surrogates of plant fitness?

## 2. Methods

### 2.1. Field Samples

**Figure 1:**
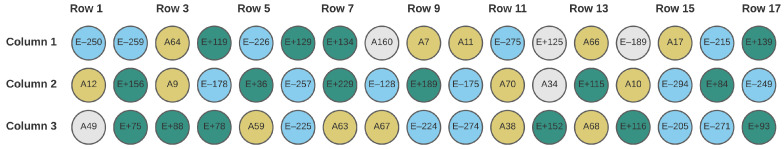
Field site layout. Plants were established in the field 30 June 2011. They were sampled for this experiment in 2017 and 2018. E− (blue) denotes plants with no *Epichloë* endophytes, E+ (green) denotes plants infected with the common toxic strain of *E. coenophiala*, and A (yellow) denotes plants infected with the AR542 strain of *E. coenophiala* (sold commercially as MaxQ). Grey circles indicate plants which died or were removed from analysis (see Methods for details).

Fifty-one tall fescue plants (cv. Georgia 5; [32]) comprising three endophyte treatments (common-toxic strain, E+; novel strain, AR542, sold commercially as MaxQ; and an *Epichloë*-free control, E−) were grown from seed in a greenhouse. Georgia 5 seed lines were originally obtained from Donald Wood (University of Georgia, USA). Ten tillers from each plant were potted in the first week of May 2011 and grown in the greenhouse until 30 June, when they were transplanted to the field at the Guelph Turfgrass Institute (Guelph, ON, Canada; 43°32′56″ N, 80°12′39″ W). Details about the climate at this field site can be found in Figure S1. The plants were arranged in a 3×17 grid and were completely randomized (see Figure 1 for field layout). Since *E. coenophiala* is a strictly vertically transmitted endophyte, there was no risk of contamination of the *Epichloë* treatment between plants [2]. The plants were watered three times a week (M,W,F) until 8 August 2011, and the area around each plant was trimmed and mowed regularly throughout the growing season. After this initial set-up period, the plants were left to grow unmanaged with the exception of occasional mowing of the area immediately surrounding each plant.

**Figure 2:**
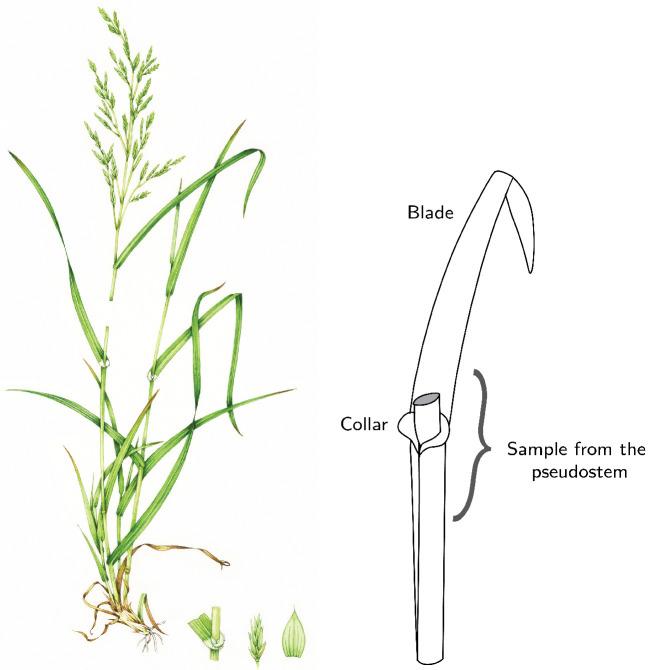
Illustration of sampled material. The colored art is used under license from Science Photo Library (https://www.sciencephoto.com, 25 September 2022 ). The line drawing is adapted from König et al. [27].

By May 2017, one E+ plant (E+125) and one AR542 plant (A160) had died, leaving 49 plants remaining. Tissue and seed samples were collected from each plant in July 2017 and 2018. An approximately 3–4 cm section of the pseudostem (see Figure 2) was collected from four tillers per plant and immediately flash frozen in liquid nitrogen, then freeze-dried and stored at −20 ${}^{\circ }C$ until further processing.

To assess plant fitness, each summer the number of tillers on each plant were counted and seed heads were collected weekly and counted in the lab.

### 2.2. DNA Extraction and Sequencing

Three out of the four tillers collected from each plant were pooled and ground in a Geno/Grinder (SPEX^®^ SamplePrep, USA). One tiller was kept as a backup in case of sample loss. DNA was extracted from 20 mg of this ground tissue using the DNeasy Plant Mini Kit (Qiagen Inc., Toronto, ON, Canada).

The fungal ITS2 region was amplified using the fITS7 (5${}^{\prime }$-GTGARTCATCGAATCTTTG-3${}^{\prime }$) and ITS4 (5${}^{\prime }$-TCCTCCGCTTATTGATATGC-3${}^{\prime }$) primers [33,34]. Each amplicon PCR reaction (25 μL) contained 2.5 μL of genomic DNA (10 ng/μL), 5 μL of each 1 μmol primer, and  12.5 μL of 2× KAPA HiFi HotStart ReadyMix (Molecular BioProducts Inc., Toronto, Canada). The PCR program was: 95 ${}^{\circ }C$ for 3 min; 25 cycles of 95 ${}^{\circ }C$ for 30 s, 55 ${}^{\circ }C$ for 30 s, and  72 ${}^{\circ }C$ for 30 s; followed by 72 ${}^{\circ }C$ for 5 min. Each index PCR reaction (50 μL) contained 5 μL of amplicon PCR product, 5 μL of each index primer, 10 μL of water, and  25 μL of 2× KAPA HiFi HotStart ReadyMix. The PCR program for the index PCR was: 95 ${}^{\circ }C$ for 3 min; 8 cycles of 95 ${}^{\circ }C$ for 30 s, 55 ${}^{\circ }C$ for 30 s, and  72 ${}^{\circ }C$ for 30 s; and 72 ${}^{\circ }C$ for 5 min.

PCR products were then purified using AMPure XP beads to remove residual primers and other PCR reagents. Paired-end sequencing (2×300bp) was performed on an Illumina MiSeq using the MiSeq Reagent Kit v3 (Illumina, San Diego, CA, USA).

Of the 49 surviving plants, 48 were sequenced (omitted A64 due to low DNA concentration). After sequencing, several samples were left out of statistical analyses (E−250-S17 due to low read count, A34 and A49 due to loss of *Epichloë* over time, and E−189 due to potentially being a mislabelled E+ plant). Due to an issue with sequencing depth in the initial sequencing run, 10 samples were re-run. Data from the original run were omitted from statistical analyses for these 10 samples, but were retained when obtaining experiment-wide ASV totals and taxonomic information.

### 2.3. Epichloë Concentration

To estimate the concentration of *E. coenophiala* in samples, quantitative PCR (qPCR) was performed on the TefA (translation elongation factor 1-α) gene using primers specific to *Epichloë* (forward primer 5${}^{\prime }$-CAATGCAGCGAGTGAACATC-3${}^{\prime }$ and reverse primer 5${}^{\prime }$-CACGTACTGACTGAAGCGTAGC-3${}^{\prime }$) on a Roche LightCycler 480 (Roche Diagnostics, Rotkreuz, Switzerland). Each reaction contained 6 μL DNA (0.5 ng/μL), 7.5 μL SYBR Green PCR mix (Roche Diagnostics, Rotkreuz, Switzerland), and  0.75 μL of each primer (at 10 μmol concentration) and each sample had three technical replicates. Each qPCR plate also included a negative control (water). The PCR program was: 95 ${}^{\circ }C$ for 5 min; 45 cycles of 95 ${}^{\circ }C$ for 10 s, 64 ${}^{\circ }C$ for 15 s, and  72 ${}^{\circ }C$ for 15 s; followed by 95 ${}^{\circ }C$ for 5 s, 65 ${}^{\circ }C$ for 1 min, continuous acquisition at 97 ${}^{\circ }C$, and  40 ${}^{\circ }C$ for 30 s to obtain the melt curve. For further details about the qPCR protocol, see Ryan et al. [35].

### 2.4. Bioinformatics

We used the DADA2 ITS Pipeline Workflow (v1.8, https://benjjneb.github.io/dada2/ITS_workflow.html, accessed on 26 September 2022 [36]). Briefly, this workflow is an ITS-specific variation of version 1.8 of DADA2. After removing primers using cutadapt (v2.3) [37], reads were filtered, trimmed, and sorted into amplicon sequence variants (ASVs). ASVs are increasingly being used in microbiome research instead of operational taxonomic units (OTUs) due to their higher resolution and consistency across studies [38]. The end product is an ASV table providing the number of times each exact ASV was observed in each sample. It also assigns taxonomy to the output using the UNITE database v8.2 [39].

### 2.5. Statistical Analyses

**Figure 3:**
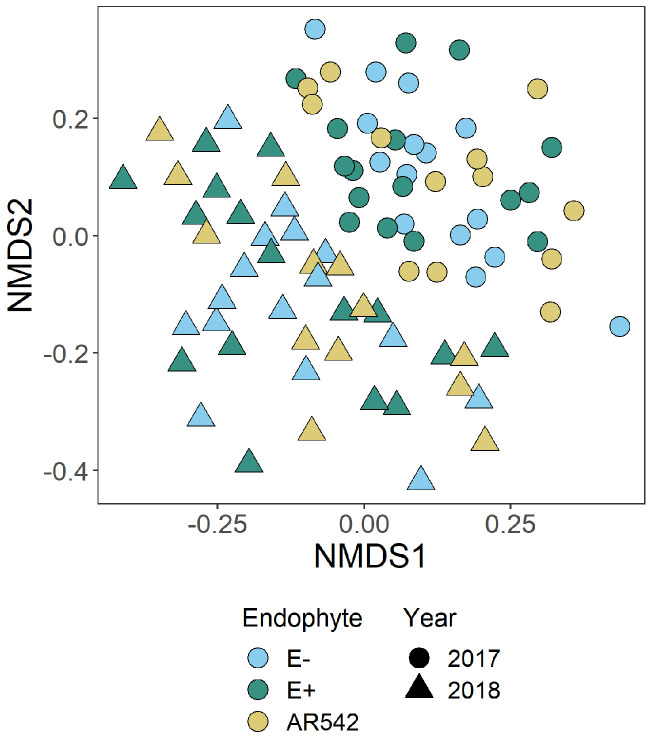
Non-metric multidimensional scaling (NMDS) analysis for all ASVs (excluding *Epichloë*). There is strong separation by year, but a great deal of overlap between the *Epichloë* treatments.

PERMANOVA (using Bray-Curtis distances) was performed on rarefied ASV data (adonis function in vegan R package [40,41] (R v4.0.1, vegan v2.5-7) to identify whether community composition differed between endophyte treatments. The pairwiseAdonis package [42] in R was used for post hoc analysis. Because non-metric multidimensional scaling (NMDS) showed strong clustering of samples by year (Figure 3), and because our data contain repeated measures (which are not supported by the adonis function), PERMANOVA was performed separately for each year.

Numerous methods exist to normalize microbiome data. One of the most common, rarefaction, has been criticized in recent years due to the potential loss of statistical power that comes from discarding data [43,44]. This is primarily an issue when looking at α-diversity or identifying differentially abundant ASVs, where other methods and transformations may be more appropriate. When comparing overall community composition between samples, rarefied data can still be clustered accurately and may in fact be one of the best normalization methods [45,46].

Differentially abundant ASVs were identified using ANCOM-BC [47]. For the 43 core ASVs, we also performed repeated measures ANOVAs on rarefied read counts.

A repeated measures ANOVA was performed on seed count, tiller count, and endophyte concentration data to test for effects of endophyte treatment and year. Sample E−250 from 2018 was removed from this analysis to maintain a balanced design (due to the corresponding 2017 sample being removed earlier due to a low read count; see Section 2.2).

We follow Wasserstein et al. [48] in reporting exact *P*-values where practical and avoiding the use of the terms “significant” and “non-significant.” Furthermore, we follow Greenland [49] by also reporting the Shannon information transformation, $s=-\log_{2}\left(P\right)$.

## 3. Results

After filtering and trimming, there were 10,357,271 sequences. From these, we obtained 3487 amplicon sequence variants (ASVs) across all samples, including two ASVs corresponding to the two strains of *E. coenophiala*. After rarefaction, we had 2165 ASVs remaining.

### 3.1. Taxonomic Variability across *Epichloë* Treatments and Years

The 10 most common taxa at each level of organization (from phylum to species) are shown in Table 1. Dothideomycetes was far more common than any other class both in terms of ASV counts (≈41% of all ASVs) and total reads (≈65% of all reads; see Table 1). Pleosporales (Dothideomycetes) was the most common order overall (≈34% of all ASVs, ≈62% of all reads). Two other common orders were Helotiales (Leotiomycetes; ≈6% of all ASVs, ≈10% of all reads) and Tremellales (Tremellomycetes; ≈5% of all ASVs, ≈3% of all reads). The most common family was Phaeosphaeriaceae (Pleosporales; ≈20% of all ASVs, ≈29% of all reads). This was followed by Corticiaceae (Corticiales; ≈3% of all ASVs, ≈3% of all reads) and Bulleribasidiaceae (Tremellales; ≈3% of all ASVs) for ASV count and Didymosphaeriaceae (Pleosporales; ≈6% of all reads) and Didymellaceae (Pleosporales; ≈6% of all reads) for total reads. The most common genus was *Septoriella* (Phaeosphaeriaceae; ≈4% of all ASVs, ≈9% of all reads) for both ASV count and total reads, followed by *Parastagonospora* (Phaeosphaeriaceae) and *Phaeosphaeria* (Phaeosphaeriaceae; ≈2% of all ASVs) for ASV count and *Paraphaeosphaeria* (Didymosphaeriaceae; ≈6% of all reads) and *Pyrenochaetopsis* (Cucurbitariaceae; ≈6% of all reads) for total reads.

### 3.2. ASV Co-Occurrences

Table 2 shows the pattern of ASV occurrences and co-occurrences (in the rarefied data) by *Epichloë* treatment and year. In Table 2, an ‘occurrence’ indicates that the ASV was present in at least one plant in the *Epichloë* × year combination. A ‘co-occurrence’ indicates that the ASV was present in at least one plant from each *Epichloë* pair or triplet. More than 70% of ASVs were present only in a single *Epichloë* treatment and single year ([264+272+232+315+264+191=1538]/2165). More than 13% of all ASVs were present in all *Epichloë* treatments and both years (302/2165). Approximately 80% of all ASVs occurred in only one year or the other but not in both years ([899+832]/2165). Together, these results suggest that a large segment of the plant’s microbiome is ‘transient.’

### 3.3. Fungal Community Structure

Non-metric multidimensional scaling (NMDS) indicated strong separation of fungal communities by year but a great deal of overlap between *Epichloë* treatments (Figure 3). This was confirmed with PERMANOVA, which showed no difference in fungal communities between *Epichloë* treatments in 2017 and a small difference in 2018 (see Table 3). Post-hoc analysis could not identify which specific pair(s) of *Epichloë* treatments were different in 2018. The PERMANOVA also indicated differences in communities depending on the location of the plant within the field site (Row and Column).

### 3.4. Draft Core Microbiome

We sought to characterize the core microbiome, which we define as those ASVs present in all or most of the plants in each *Epichloë* treatment and across both years. We refer to this as the ‘draft core microbiome’. Table 4 shows the ASVs that meet these criteria. The strictest definition—ASV must be present in all plants and all years—picked out 13 ASVs that are strong candidates for membership in the core microbiome. Our least strict definition permitted an ASV to be absent from up to five plants in any treatment by year combination. Under this definition, only 43 ASVs met the inclusion criterion. This is <2% of the total ASVs we identified and yet these 43 ASVs together comprise 69% of the (post-rarefied) total reads.

### 3.5. Guilds

We attempted to assign guilds to the ASVs that comprised the draft core microbiome. We used the FungalTraits database [50]. For the ASVs that we were able to identify to the species level (tentatively; see Section 4.5), we were only able to match two to entries in this database: ASV32 and ASV95, both of which were classified as *Epicoccum nigrum*, a plant pathogen and endophyte. Ten core ASVs could be matched at the genus level, of which seven were classified as plant pathogens on at least some of their host plants, and six were classified as endophytes in at least some of their plant hosts. For those ASVs identified to the genus level that did not match anything in FungalTraits database, we searched the CABI Invasive Species Compendium [51]. Unfortunately, the guilds for many of these ASVs are not known. Across the dataset as a whole (not only the draft core), common guilds included plant pathogens, endophytes, animal pathogens, and wood saprotrophs.

### 3.6. Differential Reads

ANCOM-BC (analysis of compositions of microbiomes with bias correction, [47]) identified several ASVs which were differentially abundant between endophyte treatments and between years. These are shown in Table 5. Between years, 48 ASVs were differentially abundant, although there is no obvious common pattern in these results (33 ASVs were more common in 2017, 15 ASVs were more common in 2018). Between *Epichloë* treatments, only 5 ASVs were differentially abundant, which is not enough to draw any conclusions.

These numbers cannot reflect some of the interesting spatial patterns that emerge from the data. In the Supplemental information (Table S2), we show 15 examples of such interesting patterns.

### 3.7. *Epichloë coenophiala* Concentrations and Plant Fitness

**Figure 4:**
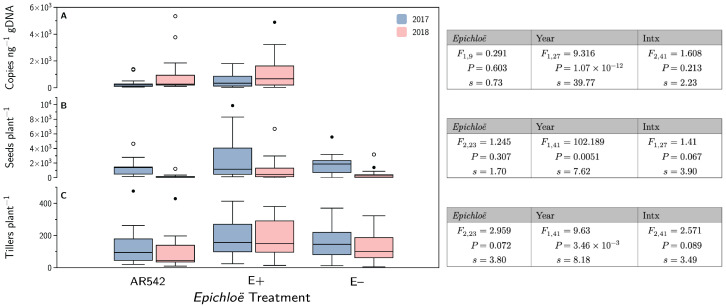
Plant reproduction and *Epichloë* concentration. Plotted are the untransformed data. (**A**) denotes the number of copies of TefA gene, used to estimate the concentration of *Epichloë* endophytes in the host plant, expressed per ng of total genomic DNA. (**B**) shows the number of seeds per plant. (**C**) shows the number of live tillers per plant. The • symbols indicate observations >1.5× the inner quartile range, and the ∘ symbols indicate observations that are >3× the inner quartile range.

The *E. coenophiala* concentrations are shown in Figure 4A. These concentrations were higher in 2018 (1109 copies ng^−1^ gDNA) than 2017 (457 copies ng^−1^ gDNA) but similar between the E+ and AR542 treatments.

Plants produced fewer seeds and tillers in 2018 (637 seeds and 137 tillers per plant) compared to 2017 (1935 seeds and 158 tillers per plant) (Figure 4B,C). Seed and tiller numbers were similar between *Epichloë* treatments.

### 3.8. Relationship between Core ASVs, Plant Fitness, and *Epichloë* Concentrations

**Figure 5:**
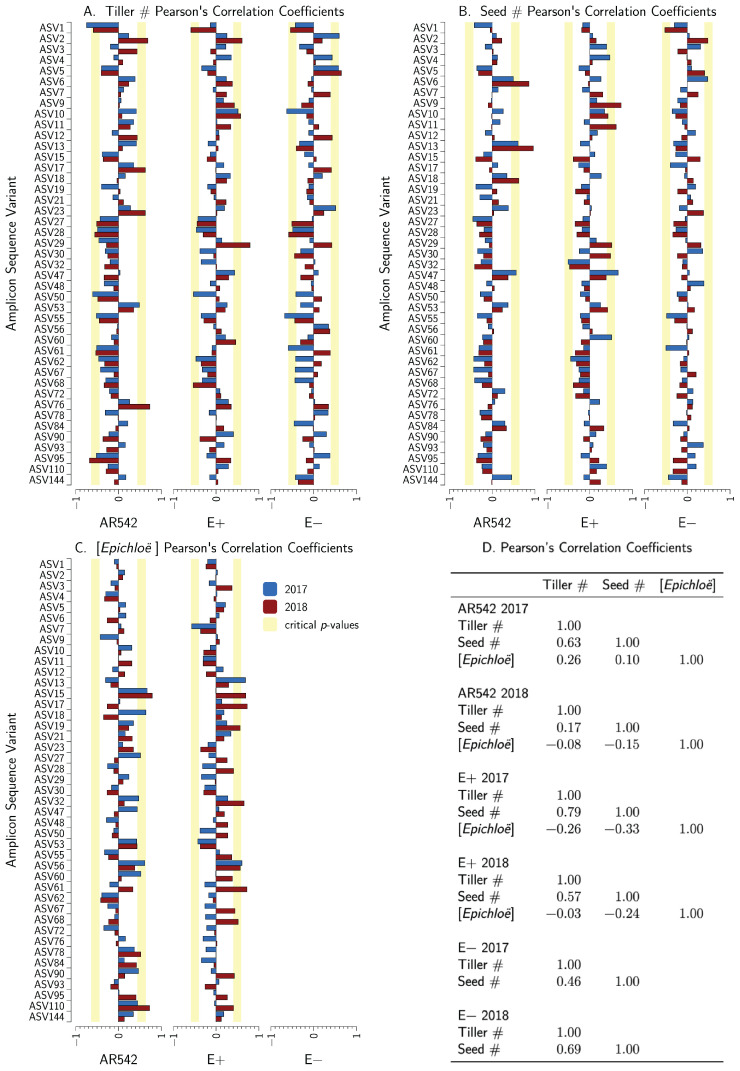
Relationship between core ASVs, plant fitness, and *Epichloë* concentrations. Blue bars denote 2017 results, red bars denote 2018 results. The vertical yellow bands denote the two-tailed critical values of *r* from p=0.1 to p=0.01. Negative values of *r* for seed and tiller number suggest the ASV is a plant antagonist and positive values of *r* suggest the ASV is beneficial to the plant. (**A**) displays the linear correlation coefficients for the draft core microbiome and tiller numbers per plant segmented by *Epichloë* treatment and year. (**B**) shows the same information but for seed numbers per plant. (**C**) displays the correlation coefficients for the draft core microbiome and *Epichloë* concentrations. (**D**) shows the correlations between tiller number, seed number, and *Epichloë* concentrations again segmented by *Epichloë* treatment and year.

We calculated the Pearson’s correlation coefficients between each of the ASVs in the draft core microbiome and the concentrations of the *Epichloë*, seed production, and tiller number for each year. The results are shown in Figure 5. These results are summarized in Table 4. For tiller numbers, five ASVs had consistently negative correlations and three had consistently positive correlations. For seed numbers, eight ASVs had consistently negative correlations while only three had consistently positive correlations. For *Epichloë* concentrations, four ASVs had consistently negative correlations, while six had consistently positive correlations.

## 4. Discussion

### 4.1. Taxonomy

Many of the most common genera are known plant pathogens (*Limonomyces*, *Parastagonospora*), and some pathogens known to be associated with tall fescue and related grasses were also found in our dataset, e.g., *Puccinia* spp. (rust) [52,53] and *Fusarium oxysporum* [54]. Based on the FungalTraits database [50], other common fungal guilds in our dataset include animal pathogens, endophytes, wood saprotrophs, and lichen parasites. There were also many ASVs for which we could not obtain detailed taxonomic information; 47% of ASVs (making up 35% of overall reads) could not be identified at the genus level. Many of these may be fungi that are difficult to culture and therefore have not been classified taxonomically.

We identified 13 ASVs that were present in every plant across both years, and 43 ASVs which were absent from five or fewer plants per treatment–year combination (Table 4). We defined these 43 ASVs as the draft “core microbiome.” Combined, these core ASVs make up nearly 70% of total reads. The vast majority of core ASVs were ascomycota (38/43), and more than half were dothidiomycetes (27/43). Of those with guild information from FungalTraits available, many were potential plant pathogens. This is consistent with our plant fitness data, which indicate a negative correlation between many core ASVs and seed and tiller counts (Figure 5 and Table 4). Further work is required to see whether these particular ASVs are geographically specific and whether they persist over longer periods of time.

### 4.2. Effect of *Epichloë* and Comparisons with Previous Research

**Figure 6:**
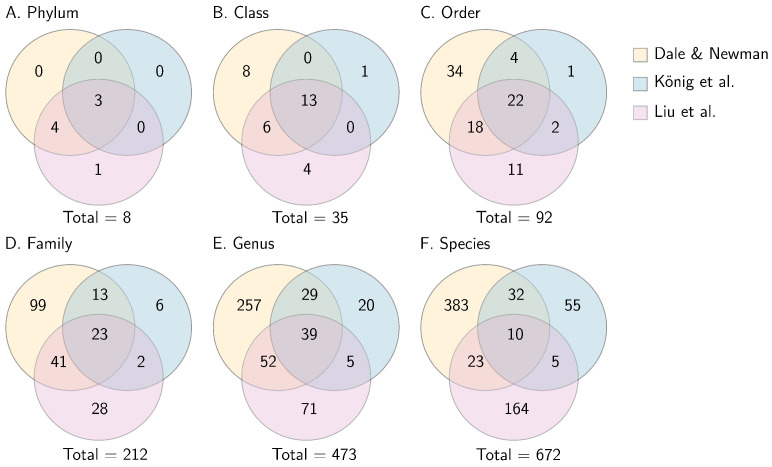
Comparison of the taxonomic diversity found in our study with that found in König et al. [27] and Liu et al. [28]. The Venn Diagrams show unique and shared numbers of phyla (**A**), classes (**B**), orders (**C**), families (**D**), genera (**E**) and species (**F**). It is clear that the overlap in diversity between the three studies is low. In (F), the 10 species that are common to all three studies are: *Alternaria rosae*, *Cystofilobasidium macerans*, *Filobasidium magnum*, *Zymoseptoria verkleyi*, *Sporobolomyces roseus*, *Dioszegia hungarica*, *Buckleyzyma aurantiaca*, *Rhodotorula babjevae*, *Malassezia restricta*, *Blumeria graminis*.

We observed diverse fungal communities in tall fescue. Of the ASVs we were able to obtain taxonomic information for, a relatively small proportion matched the taxa found in König et al. [27] and Liu et al. [28] (see Figure 6). This is perhaps not surprising given the different species (König et al. used *L. perenne*–*Epichloë festucae* var. *lolii*, Liu et al. used *A. inebrians*–*E. gansuensis*), different locations (König et al. in Germany, Liu et al. in China), and somewhat different tissue samples (König et al.. used a segment of basal leaf, Liu et al. used the 3rd and 4th leaf blades, and we used the pseudostem). Both of these studies also sequenced the ITS1 region rather than ITS2, although research has shown that results from both regions are comparable [55]. Finally, both of these studies also appear to exclude low-abundance OTUs, whereas we retained any ASVs with more than two reads, which likely explains why we observed the largest number of unique taxa. Nissinen et al. [26] looked at fungal communities in tall fescue, though as of writing the full data set is not available for comparison. Of their 11 most common taxa (excluding *Epichloë*; see Figure 1 in Nissinen et al.), two species were found in our data set (*Puccinia coronata*, *Blumeria graminis*; note however that we cannot be certain about species identification from molecular data alone, as discussed in Section 4.5). Of the remaining nine taxa, six were members of genera observed in our data (*Pyrenophora*, *Podospora*, *Pyrenophora*, *Cryptococcus*, *Eutypa*, *Colletotrichum*), and two were members of families observed in our data (Nectriaceae, Glomerellaceae).

Presence of *Epichloë* did not consistently alter the fungal community composition, but there was some difference in 2018 when *Epichloë* concentration was highest. *Epichloë* have been found to have antifungal properties before. However, these results may be due to factors other than the alkaloids that confer many of its other effects, because Fernando et al. [56] found no antifungal activity for a variety of common *Epichloë*-produced alkaloids on several plant pathogens.

Research to date on the effect of *Epichloë* on fungal communities has been variable. König et al. [27] and Liu et al. [28] observed no effect of *Epichloë festucae* var. *lolii* and *Epichloë gansuensis* on the fungal microbiome of perennial ryegrass and drunken horse grass, respectively, whereas Nissinen et al. [26] found an effect of *E. coenophiala* on tall fescue fungal microbiomes. Using a different approach, Zabalgogeazcoa et al. [57] observed no effect of *Epichloë festucae* (M.J. Latch Chr. & Samuels) C.W. Bacon & Schardl on the *culturable* non-systemic fungal microbiomes of red fescue (*Festuca rubra* L.).

Several ASVs in our study were differentially abundant between *Epichloë* treatments (see Table 5). Only one could be identified to the genus level (*Limonomyces*), which was absent from E+ plants. Previous research has shown some antifungal activity by *E. coenophiala* (and related *Epichloë* species) against *Limonomyces roseipellis* (pink patch), a fungal pathogen in grasses [58].

### 4.3. Variation over Time

There was a noticeable difference in fungal communities between the the two years measured, both in the overall communities (Figure 3) and in specific differentially abundant ASVs (Table 5). This suggests that at least some of the fungal community of tall fescue is transient. This is consistent with previous research showing that phyllosphere (leaf surface) microbial communities are strongly affected by environmental changes like temperature and moisture [59,60]. According to historical climate data from a local weather station, the mean (±standard deviation) temperatures in the month leading up to sample collection were 17.87 ± 3.20 ${}^{\circ }C$ in 2017 and 18.70 ± 3.96 ${}^{\circ }C$ in 2018, and the relative humidity values were 74.13 ± 9.36 %RH in 2017 and 70.67 ± 9.88 %RH in 2018. For more detailed climate data see Figure S1 in the Supplemental Information.

Although communities fluctuated a great deal over time, we also observed some ASVs that were consistently associated with one or more specific plants across both years (see Figure S3 in Supplementary Information).

### 4.4. Plant Fitness

*Epichloë* infection was not associated with a change in either seed count or tiller count (see Figure 4). Previous research into the effect of *Epichloë* on plant fitness has produced mixed results; although many demonstrate a positive correlation between the two [61,62,63,64], other research has also shown no *Epichloë* effect [65,66] or even a negative association between *Epichloë* infection and fitness [67]. Fitness was, however, different between the two years measured. Both seed counts and tiller numbers were higher in 2017. These results may be due to environmental differences such as herbivore pressure, temperature or precipitation.

### 4.5. Limitations

The strong differences in fungal communities between the two years suggest that these communities fluctuate over time, so it would be interesting to observe them over more years to obtain a clearer longitudinal picture. Additionally, because we performed DNA extraction on the 2017 and 2018 samples at different times, it is possible that some of the changes observed between the two years could be due to this rather than true biological differences. However, the differences we observed in tiller and seed numbers between years suggests that there was something different between the years, and climate data in Section 4.3 suggests one possible source of this difference.

The taxonomic information included in this paper is limited to what is currently available in databases; many of the fungi that have been cultured and described are plant pathogens, and therefore our results may be biased towards these fungi. Therefore, caution is warranted in interpreting these guild data. Additionally, although we provide species-level identities for some ASVs, these are only tentative; without more information (such as sequences from additional barcode regions) we cannot be completely confident about species assignment from the ITS2 region alone [68].

### 4.6. Conclusions and Future Directions

Overall, the fungal communities we observed in tall fescue were diverse but not strongly affected by the presence of *E. coenophiala*. Although some fungi appeared to have a long term association with some or most plants, most were rare, and seemed to have a more transient relationship with their host as shown by the strong differences in the communities between years. In the future it would be interesting to observe these communities over a longer time period. It also might be helpful to investigate phyllosphere and endosphere communities separately, given that phyllosphere communities are likely to be more transient in nature than endophytic communities.

This research provides an initial assessment of the composition of the tall fescue fungal microbiome, but relatively little about how it might function in the plant. Next steps might include using other “omics” technologies (transcriptomics, proteomics, metabolomics) to clarify some of these mechanisms. Future research might also involve the use of plants that are genetic clones treated with a dilution series of a systemic fungicide (analogous to a gene knockout experiment) to further assess the fitness consequences of the fungal microbiome in this species.   

## Figures and Tables

**Table 1:** Most common taxonomic groups by amplicon sequence variants (ASVs) occurences or the number of reads, using either the raw data or the rarefied data. In each case the proportions of the totals are shown. For each taxonomic category and measure of abundance (columns), the most abundant taxon is highlighted in blue-green, the second most abundant is highlighted in rose and the third most abundant is highlighted in yellow. Note that species identities listed here are only tentative; see Section 4.5.

Phylum (P), Class (C), Order(O)	Family	Genus	Species	ASVs Raw	ASVs Rarefied	Reads Raw	Reads Rarefied
P: Ascomycota				0.680	0.670	0.896	0.895
C: Dothideomycetes				0.418	0.411	0.662	0.649
O: Capnodiales				0.037	0.039	0.047	0.041
	Cladosporiaceae	*Cladosporium*	*cladosporioides*			0.017	0.018
	Neodevriesiaceae	*Neodevriesia*	*poagena*	0.004	0.005		
	Teratosphaeriaceae			0.011			
O: Pleosporales				0.352	0.343	0.633	0.618
	Cucurbitariaceae					0.059	0.062
	Cucurbitariaceae	*Pyrenochaetopsis*				0.059	0.062
		*Pyrenochaetopsis*	*leptospora*			0.036	0.038
	Coniothyriaceae	*Coniothyrium*	*crepinianum*	0.003			
	Didymellaceae			0.017	0.020	0.060	0.074
		*Neoascochyta*				0.049	0.062
		*Neoascochyta*	*tardicrescens*			0.049	0.060
	Didymosphaeriaceae				0.012	0.062	0.061
		*Paraphaeosphaeria*				0.062	0.061
		*Paraphaeosphaeria*	*rosae*			0.014	0.012
	Lentitheciaceae			0.021	0.020	0.030	0.034
		*Keissleriella*		0.009	0.010		
		*Keissleriella*	*quadriseptata*		0.004		
	Phaeosphaeriaceae			0.204	0.188	0.307	0.275
		*Neosetophoma*	*samararum*	0.004			
		*Parastagonospora*		0.0253	0.244	0.0396	0.0299
		*Phaeosphaeria*		0.017	0.019		
		*Piniphoma*	*wesendahlina*			0.019	0.020
		*Septoriella*		0.044	0.032	0.091	0.085
		*Septoriella*	*phragmitis*	0.042	0.031	0.092	0.087
	Pleosporaceae			0.015	0.017	0.028	0.028
		*Alternaria*		0.009	0.011	0.028	0.028
		*Alternaria*	*infectoria*	0.005	0.006		
C: Eurotiomycetes				0.028	0.029	0.023	0.023
O: Chaetothyriales				0.027	0.028	0.023	0.023
C: Lecanoromycetes				0.016	0.018	0.064	0.063
O: Ostropales					0.013	0.063	0.063
	Gomphillaceae					0.043	0.042
		*Corticifraga*				0.043	0.041
		*Corticifraga*	*peltigerae*		0.004	0.043	0.041
	Stictidaceae	*Neofitzroyomyces*				0.020	0.021
		*Neofitzroyomyces*	*nerii*	0.005	0.006	0.020	0.021
C: Leotiomycetes				0.057	0.064	0.093	0.108
O: Helotiales				0.053	0.061	0.093	0.108
	Helotiaceae			0.053	0.061	0.093	0.108
		*Articulospora*		0.012	0.014	0.020	
		*Articulospora*	*proliferata*	0.012	0.014	0.020	0.020
	Hyaloscyphaceae			0.012	0.015		
C: Saccharomycetes				0.012			
O: Saccharomycetales				0.012			
	Metschnikowiaceae	*Metschnikowia*		0.011			
C: Sordariomycetes				0.072	0.075	0.046	0.042
O: Glomerellales						0.020	0.017
O: Hypocreales				0.029	0.030	0.014	0.016
O: Xylariales						0.007	
P: Basidiomycota				0.254	0.272	0.100	0.100
C: Agaricomycetes				0.112	0.119	0.059	0.055
O: Agaricales				0.036	0.038		
	Psathyrellaceae			0.011	0.012		
O: Cantharellales				0.012			0.005
O: Corticiales				0.032	0.039	0.047	0.041
	Corticiaceae			0.032	0.038	0.047	0.041
		*Laetisaria*			0.011		
		*Laetisaria*	*lichenicola*	0.009	0.011		
		*Limonomyces*		0.014	0.017	0.032	0.032
C: Cystobasidiomycetes				0.022	0.023	0.003	0.003
C: Microbotryomycetes				0.012	0.013	0.002	0.002
C: Tremellomycetes				0.065	0.077	0.034	0.038
O: Tremellales				0.049	0.056	0.028	0.032
	Bulleribasidiaceae			0.030	0.034	0.025	0.029
		*Dioszegia*		0.010	0.012		
		*Vishniacozyma*		0.014	0.016		0.022
		*Vishniacozyma*	*dimennae*	0.004	0.004		
		*Vishniacozyma*	*tephrensis*			0.010	0.012
		*Vishniacozyma*	*victoriae*	0.004	0.004		
P: Chytridiomycota				0.013	0.014	0.003	0.003
C: Spizellomycetes					0.013	0.003	0.003
O: Spizellomycetales					0.013		
P: Mortierellomycota				0.002	0.001	0.000	0.000
P: Mucoromycota				0.000	0.000	0.000	0.000
P: Olpidiomycota				0.000		0.000	0.000
P: Rozellomycota				0.000		0.000	0.000

**Table 2:** ASV occurrences and co-occurrences (in rarefied data) by *Epichloë* treatment and year.

*Epichloë* Treatment	2017 Only	2018 Only	Both Years	Subtotal
E+ only	264	315	33	612
E− only	272	264	45	581
AR542 only	232	191	24	447
E+, E− only	42	15	15	72
E+, AR542 only	27	20	6	53
E−, AR542 only	32	11	9	52
E+, E−, AR542	30	16	302	348
Subtotal	899	832	434	2165

**Table 3:** PERMANOVA analyses by year. The row and column sources of variance refer to the physical layout of the experiment, see Figure 1. df= degrees of freedom, SS= sum of squares, MS= mean squares, F= pseudo-*F* statistic, P= *p*-value; *s* denotes the Shannon Information Transformation ($s=-\log_{2}\left(p\right)$); see Section 2.5. The *Epichloë* treatment was important in 2018 but not 2017.

A. 2017							
Source	*df*	*SS*	*MS*	*F*	$R^{2}$	*P*	*s*
*Epichloë*	2	0.34	0.17	1.04	0.04	0.3873	1.37
Row	16	3.15	0.20	1.20	0.41	0.0144	6.12
Column	2	0.45	0.236	1.37	0.06	0.0349	4.84
Residuals	23	3.78	0.16		0.49		
Total	43	7.72			1.00		
**B. 2018**							
Source	*df*	*SS*	*MS*	*F*	$R^{2}$	*P*	*s*
*Epichloë*	2	0.39	0.19	1.42	0.05	0.0453	4.46
Row	16	3.03	0.19	1.39	0.42	0.0007	10.48
Column	2	0.48	0.24	1.76	0.07	0.0061	7.35
Residuals	23	3.26	0.14		0.46		
Total	43	7.16			1.00		

**Table 4:** Amplicon Sequence Variants (ASVs) that may comprise the core microbiome. Note that taxonomic assignments at the species level are only tentative; see Section 4.5. In the *Epichloë* treatment columns, the number of plants containing the ASV are shown. The maximum number of plants that may harbour an ASV are: AR542: N=13, E−: N=15 in 2017 and N=16 in 2018, E+: N=16. The deeper red shading indicates more plants are missing the ASV. ASVs are grouped by the maximum number of plants that are missing that particular ASV, from zero—i.e., the ASV is present in all plants and in both years—to five, where the ASV is missing from five or fewer plants in *at least* one *Epichloë* treatment × year combination. The three columns showing *p*-values are from a repeated measures ANOVA conducted on the number of reads (Intx denotes the interaction of *Epichloë* and year). Values in purple denote that the *p*-values are below the Benjamini–Hochberg Procedure critical value for controlling False Discovery Rate. Missing entries in the *p*-value columns indicate that the data could not be adequately transformed to meet the assumptions of the test. Note that these 43 ASVs account for 69% of all reads across both years. Consistent correlations denote ASVs that have consistent correlations (positive or negative) across all *Epichloë* treatments and years. For tiller and seed numbers, r<0 suggest these ASVs are plant antagonists, and r>0 suggest the ASVs are beneficial to the plant. For the *Epichloë* concentrations, r<0 suggest a antagonistic relationship while values of r>0 suggest a mutualistic relationship. ASVs denoted in brown are thought to be plant pathogens (see Guild column). For guild information from Fungal Traits [50], E denotes endophyte, PP denotes plant pathogen, AP denotes animal pathogen, and WS denotes wood saprophyte. For those ASVs identified to genus level, that did not match anything in FungalTraits database, we searched the CABI Invasive Species Compendium [51]. From this database, gpp denotes the genus contains plant pathogens (in this case *Parastagonospora*), and pp denotes plant pathogen.

			2017 Plants			2018 Plants			Proportion	*Epichloë*	Year	Intx	Consistent Correlations			
	Taxonomic Identity	ASV	AR542	E+	E−	AR542	E+	E−	Reads	*p*-Value	*p*-Value	*p*-Value	Tiller #	Seed #	[*Epichloë*]	Guild
=0 plants	family: Phaeosphaeriaceae	ASV1	13	15	16	13	16	16	0.095	3.04 × 10${}^{-1}$	1.11 × 10${}^{-14}$	9.64 × 10${}^{-1}$	r<0	r<0	r<0	
	family: Phaeosphaeriaceae	ASV2	13	15	16	13	16	16	0.082	4.55 × 10${}^{-2}$	9.80 × 10${}^{-8}$	7.37 × 10${}^{-1}$	r>0	r>0		
	family: Didymosphaeriaceae	ASV3	13	15	16	13	16	16	0.057	8.27 × 10${}^{-1}$	1.06 × 10${}^{-1}$	3.89 × 10${}^{-1}$				
	order: Pleosporales	ASV4	13	15	16	13	16	16	0.057	5.31 × 10${}^{-1}$	2.39 × 10${}^{-7}$	2.33 × 10${}^{-1}$		r>0		
	*Neoascochyta tardicrescens*	ASV5	13	15	16	13	16	16	0.049	6.80 × 10${}^{-1}$	1.94 × 10${}^{-4}$	9.22 × 10${}^{-1}$			r>0	
	*Corticifraga peltigerae*	ASV6	13	15	16	13	16	16	0.039	2.07 × 10${}^{-2}$	9.99 × 10${}^{-3}$	5.81 × 10${}^{-1}$				
	*Pyrenochaetopsis leptospora*	ASV7	13	15	16	13	16	16	0.036	7.71 × 10${}^{-1}$	4.86 × 10${}^{-3}$	7.61 × 10${}^{-1}$				
	family: Lentitheciaceae	ASV9	13	15	16	13	16	16	0.027	5.00 × 10${}^{-1}$	7.65 × 10${}^{-1}$	6.05 × 10${}^{-1}$				
	*Volucrispora graminea*	ASV10	13	15	16	13	16	16	0.026	7.01 × 10${}^{-1}$	4.09 × 10${}^{-2}$	9.54 × 10${}^{-1}$				
	genus: *Pyrenochaetopsis*	ASV11	13	15	16	13	16	16	0.024	8.45 × 10${}^{-1}$	2.51 × 10${}^{-2}$	8.04 × 10${}^{-1}$				
	*Cladosporium cladosporioides*	ASV15	13	15	16	13	16	16	0.018	6.21 × 10${}^{-2}$	7.86 × 10${}^{-14}$	5.40 × 10${}^{-1}$				E,PP,WS
	order: Chaetothyriales	ASV19	13	15	16	13	16	16	0.014	4.20 × 10${}^{-1}$	5.92 × 10${}^{-1}$	6.93 × 10${}^{-1}$			r>0	
	*Epicoccum nigrum*	ASV32	13	15	16	13	16	16	0.006	4.58 × 10${}^{-1}$	4.15 × 10${}^{-1}$	7.52 × 10${}^{-1}$		r<0	r>0	E,PP
≤1 plant	genus: *Colletotrichum*	ASV12	13	14	16	13	16	16	0.015	8.58 × 10${}^{-1}$	6.27 × 10${}^{-12}$	6.65 × 10${}^{-1}$				E,PP
	genus: *Parastagonospora*	ASV18	13	15	15	13	15	16	0.012	5.54 × 10${}^{-1}$	5.56 × 10${}^{-1}$	3.88 × 10${}^{-3}$				
	*Alternaria rosae*	ASV21	13	15	15	13	16	16	0.011	4.85 × 10${}^{-1}$	2.25 × 10${}^{-11}$	6.46 × 10${}^{-1}$			r>0	AP,E,PP,WS
	*Alternaria alternata*	ASV27	13	15	15	13	16	16	0.008	4.00 × 10${}^{-1}$	4.64 × 10${}^{-4}$	8.69 × 10${}^{-1}$	r<0	r<0		AP,E,PP,WS
	*Vishniacozyma tephrensis*	ASV29	13	15	16	12	16	16	0.008	7.33 × 10${}^{-2}$	2.00 × 10${}^{-16}$	7.25 × 10${}^{-2}$				
	genus: *Parastagonospora*	ASV28	13	14	15	13	16	16	0.007	6.26 × 10${}^{-1}$	1.18 × 10${}^{-2}$	4.15 × 10${}^{-1}$	r<0	r<0		
	*Vishniacozyma tephrensis*	ASV61	13	15	16	12	16	15	0.003							
	family: Tubeufiaceae	ASV76	13	15	15	13	16	15	0.002	8.09 × 10${}^{-1}$	2.61 × 10${}^{-5}$	6.42 × 10${}^{-1}$	r>0			
≤2 plants	*Vishniacozyma victoriae*	ASV50	13	15	16	11	16	15	0.004	3.37 × 10${}^{-1}$	8.44 × 10${}^{-8}$	3.29 × 10${}^{-2}$		r<0		
	*Phialophora livistonae*	ASV62	11	15	15	12	16	15	0.003	3.88 × 10${}^{-2}$	4.85 × 10${}^{-2}$	2.92 × 10${}^{-1}$		r<0	r<0	
	*Devriesia pseudoamericana*	ASV67	12	15	14	11	16	15	0.002							
	order: Pleosporales	ASV68	11	13	15	12	15	14	0.002	2.93 × 10${}^{-1}$	4.81 × 10${}^{-1}$	9.01 × 10${}^{-1}$	r<0	r<0		
	*Epicoccum nigrum*	ASV95	11	14	16	12	14	15	0.001	9.93 × 10${}^{-1}$	1.95 × 10${}^{-2}$	3.61 × 10${}^{-1}$				E,PP
≤3 plants	family: Phaeosphaeriaceae	ASV17	13	14	15	11	13	13	0.011	2.90 × 10${}^{-1}$	3.51 × 10${}^{-6}$	9.22 × 10${}^{-1}$				
	*Articulospora proliferata*	ASV23	12	15	15	13	13	14	0.010	1.78 × 10${}^{-1}$	1.61 × 10${}^{-3}$	4.30 × 10${}^{-1}$	r>0			
	*Alternaria infectoria*	ASV47	10	15	13	12	13	15	0.004							AP,E,PP,WS
	genus: Cyphellophora	ASV53	10	15	15	10	13	15	0.003					r>0		AP
	*Paraophiobolus plantaginis*	ASV144	10	12	13	10	15	14	0.001						r>0	
≤4 plants	*Piniphoma wesendahlina*	ASV13	10	11	12	12	12	13	0.020							
	genus: Cryptocoryneum	ASV48	9	11	13	12	12	15	0.004							
	*Alternaria infectoria*	ASV60	11	13	12	12	16	16	0.003							pp
	*Vishniacozyma dimennae*	ASV84	13	15	16	9	13	14	0.002	3.47 × 10${}^{-1}$	1.03 × 10${}^{-10}$	2.61 × 10${}^{-1}$				
	genus: *Acremonium*	ASV93	12	13	13	13	12	14	0.001							
≤5 plants	*Neofitzroyomyces nerii*	ASV30	10	10	13	9	12	13	0.008				r<0		r<0	
	*Fusarium langsethiae*	ASV56	9	14	11	10	15	16	0.004						r>0	AP,E,PP,WS
	genus: *Parastagonospora*	ASV55	13	12	11	12	14	13	0.003					r<0		gpp
	family: Lentitheciaceae	ASV78	12	14	15	10	11	11	0.003							
	*Articulospora proliferata*	ASV72	9	15	12	9	11	14	0.002						r<0	
	*Paraphaeosphaeria michotii*	ASV90	8	14	11	13	16	15	0.002							pp
	*Chrysozyma griseoflava*	ASV110	11	13	15	11	13	11	0.001							
								sum:	0.691							

**Table 5:** Differentially abundant ASVs and the number of reads (summed across all infected plants) and the number of infected plants in which each ASV is present (by *Epichloë* treatment and Year). Highlighted ASVs are members of the draft core microbiome, see Table 4. Note that taxonomic assignments at the species level are only tentative; see Section 4.5.

		Different across *Epichloë* Treatment						Different across Years			
ASV	Taxonomy	E−		E+		AR542		2017		2018	
		Reads	Plants	Reads	Plants	Reads	Plants	Reads	Plants	Reads	Plants
ASV1	Family: Phaeosphaeriaceae							19,623	44	82,628	45
ASV2	*Septoriella phragmitis*							30,098	44	57,643	45
ASV12	*Genus: Colletotrichum*							1589	43	14,207	45
ASV15	*Cladosporium cladosporioides*							5562	44	13,512	45
ASV17	Family: Phaeosphaeriaceae							9803	43	2097	39
ASV21	*Alternaria rosae*							2777	43	9361	45
ASV29	*Vishniacozyma tephrensis*							6888	44	1502	44
ASV41	Order: Pleosporales							304	17	3072	36
ASV46	*Genus: Dioszegia*							3839	44	177	31
ASV50	*Vishniacozyma victoriae*							3179	44	1290	42
ASV60	*Alternaria infectoria*							1096	36	2431	44
ASV61	*Vishniacozyma tephrensis*							2667	44	1075	43
ASV70	*Colletotrichum eleusines*							152	15	942	30
ASV78	Family: Lentitheciaceae							2263	41	432	34
ASV84	*Vishniacozyma dimennae*							1591	44	402	37
ASV88	Order: Pleosporales							1157	42	348	32
ASV90	*Paraphaeosphaeria michotii*							581	34	1047	44
ASV98	*Ramularia collo-cygni*							392	30	805	43
ASV108	*Alfaria ossiformis*							223	17	988	34
ASV114	*Cystofilobasidium macerans*							1340	42	9	6
ASV125	*Coniothyrium crepinianum*							31	3	834	18
ASV137	*Dioszegia rishiriensis*							930	40	74	19
ASV142	*Genus: Cryptococcus*							572	39	116	19
ASV145	*Taphrina tormentillae*							747	33	94	19
ASV149	*Dioszegia rishiriensis*							686	40	132	19
ASV158	*Vishniacozyma victoriae*							669	26	42	6
ASV160	Order: Hypocreales							110	27	548	38
ASV169	Order: Erythrobasidiales							490	34	121	21
ASV178	*Genus: Dioszegia*							902	38	17	5
ASV195	*Filobasidium magnum*							520	32	89	17
ASV196	*Zymoseptoria verkleyi*							54	18	342	32
ASV197	Class: Cystobasidiomycetes							385	34	84	12
ASV227	*Genus: Limonomyces*	213	7	0	0	135	6				
ASV234	*Filobasidium stepposum*							514	28	18	10
ASV237	*Papiliotrema frias*							485	32	32	12
ASV249	Class: Lecanoromycetes	100	6	77	5	0	0				
ASV260	*Genus: Filobasidium*							282	29	63	12
ASV272	Kingdom: Fungi							168	28	34	13
ASV273	*Filobasidium oeirense*							245	26	9	4
ASV281	*Genus: Phaeosphaeria*							49	10	193	28
ASV283	*Protomyces inouyei*							324	24	0	0
ASV288	Order: Holtermanniales							283	28	38	6
ASV289	*Dioszegia rishiriensis*							151	18	18	4
ASV298	*Genus: Parastagonospora*							29	4	181	21
ASV301	*Filobasidium wieringae*							259	29	15	5
ASV318	*Sporobolomyces roseus*							179	28	1	2
ASV323	Order: Microstromatales							142	22	30	2
ASV367	Order: Pleosporales							18	3	117	20
ASV387	*Genus: Phaeosphaeria*							21	4	94	17
ASV533	Family: Extremaceae	33	8	6	3	0	0				
ASV552	*Apenidiella strumelloidea*							3	2	40	13
ASV837	Phylum: Ascomycota	6	6	7	4	0	0				
ASV875	Phylum: Ascomycota	13	8	0	0	0	3				

## Data Availability

The data presented in this study are available on Dryad at https://doi.org/10.5061/dryad.fj6q573z8 (accessed on 27 September 2022).

## Supplementary Materials

The following are available online at https://www.mdpi.com/article/10.3390/jof8101026/s1, Figure S1: Climate data, Figure S2: Rarefaction curves for all samples, Figure S3: Field maps of 15 ASVs with similar abundance patterns across years.
