# Supplementary material for: A First Draft of the Core Fungal Microbiome of Schedonorus arundinaceus with and without Its Fungal Mutualist Epichloë coenophiala
Source: J Fungi (Basel). 2022 Sep 28;8(10):1026. doi: 10.3390/jof8101026 (PMC9605371; doi:10.3390/jof8101026)
Supplement: Supplementary file 1 [file jof-08-01026-s001.zip › jof-1889084-supplementary.pdf]

Figure S1: Climate data. Shown are the data from an Environment and Climate Change Canada weather station located 18.5 km from our field site, at 43° 32" N, 80° 42" W. We sampled on 5 July 2017 and 6 July 2018, shown in C.

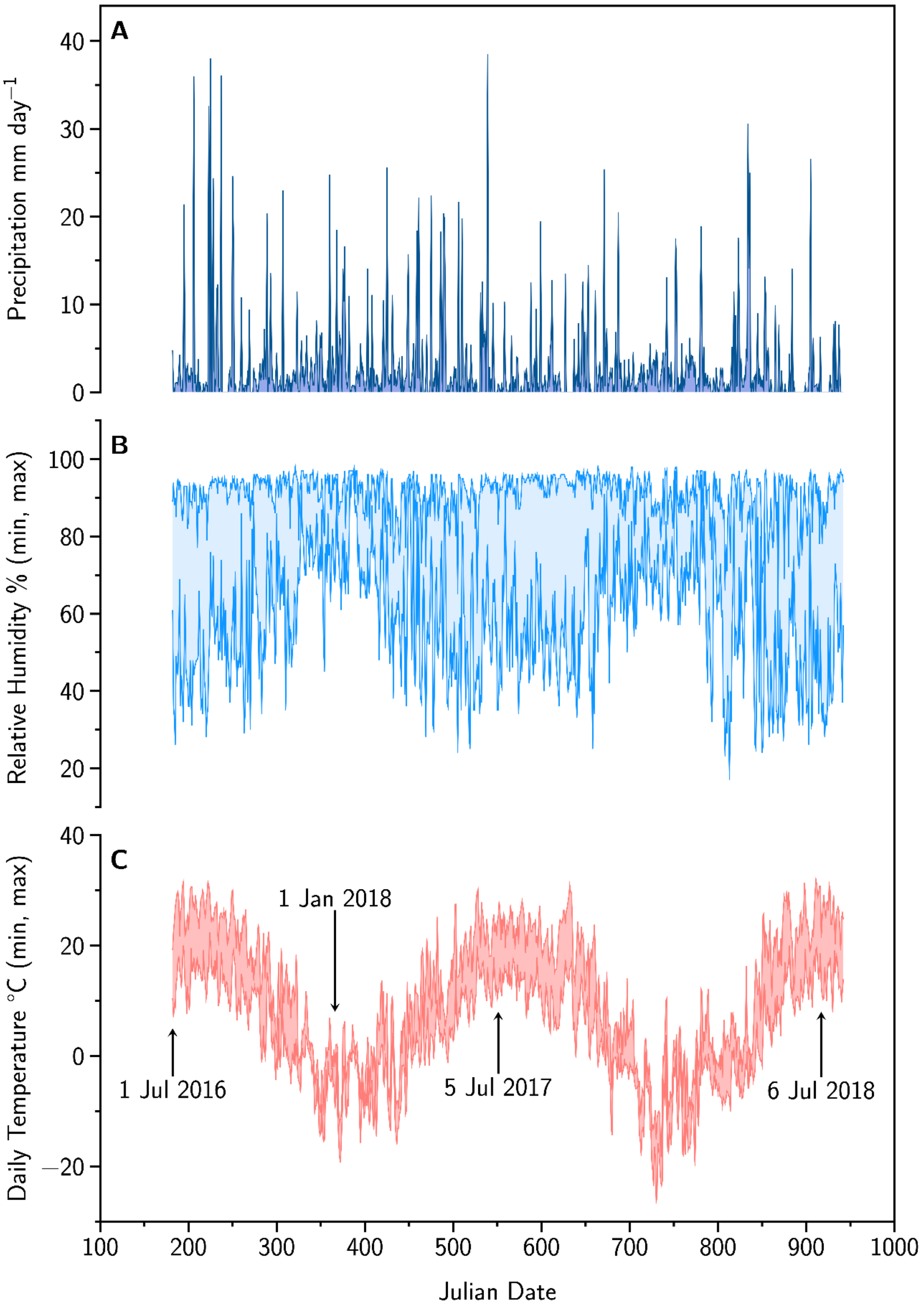

Figure S2. Rarefaction curves of ASVs and number of reads for all samples, separated by *Epichloë coenophiala* treatment and year. E+: common toxic strain; AR542: novel strain; E-: *Epichloë*-free control. Curves plateau for most samples, suggesting adequate sequencing depth.

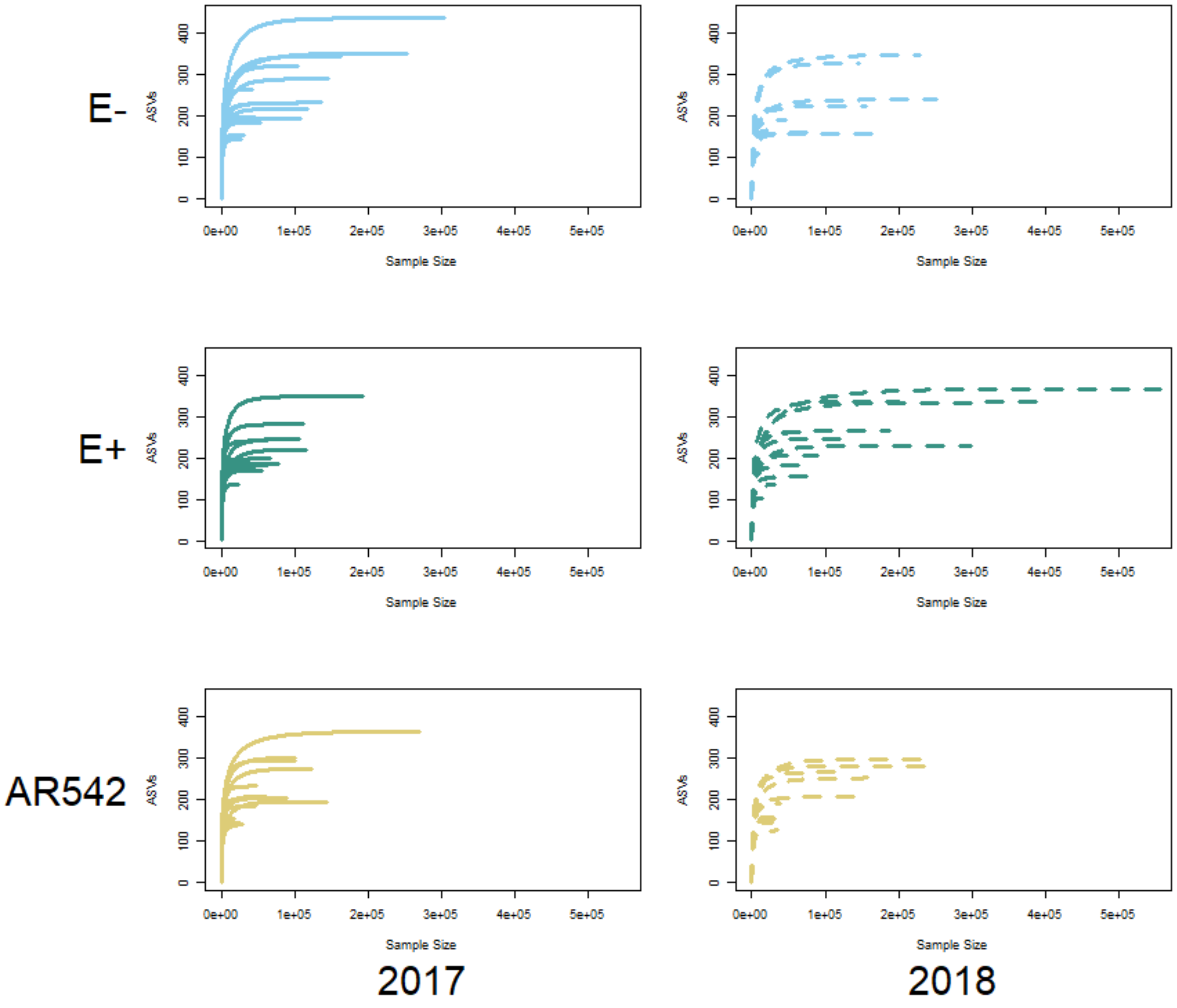

Figure S3. Field maps of 15 ASVs with similar abundance patterns across years. For plant ID information, see Figure 1 in the main text.

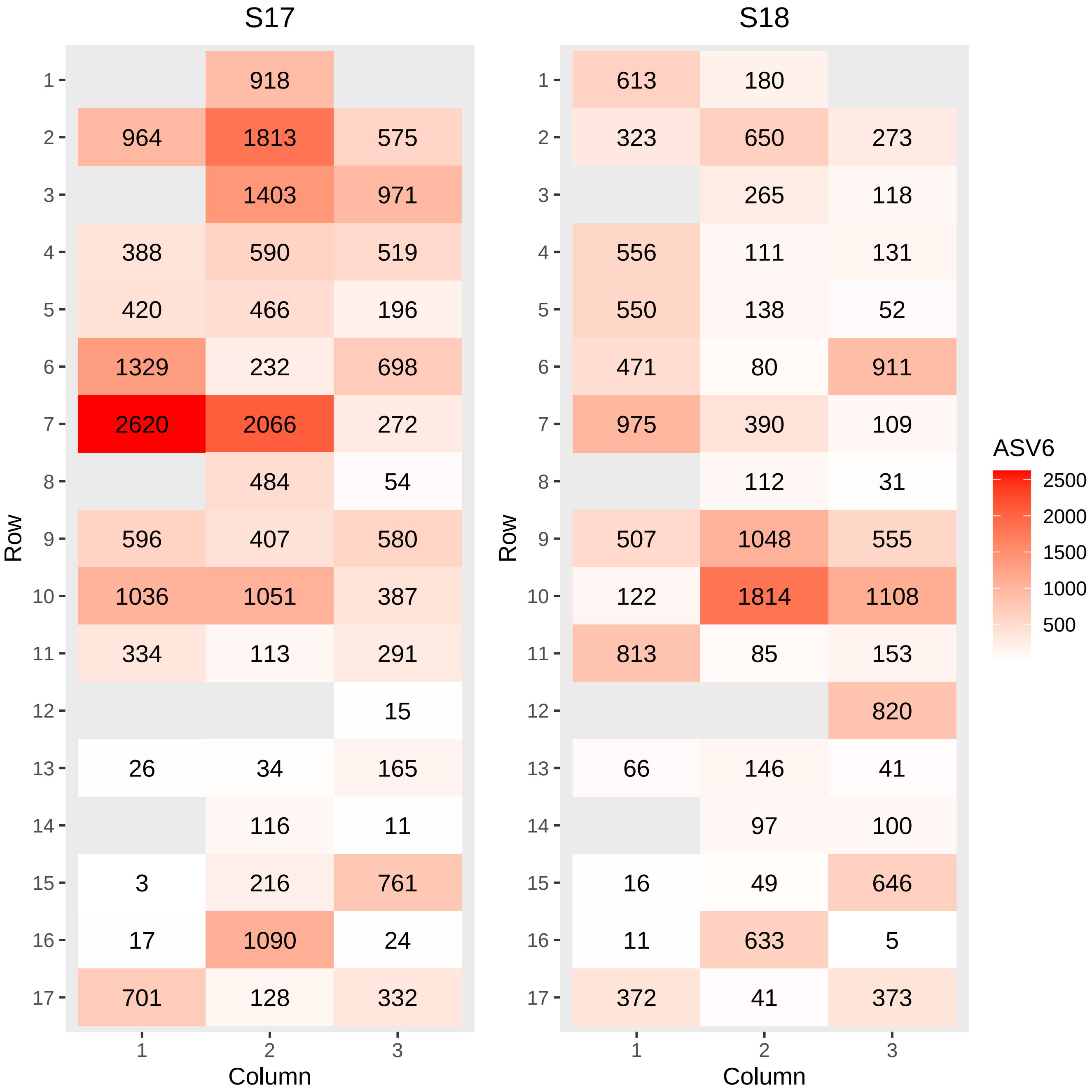
S17
S18
Row
Column
ASV6
2500
2000
1500
1000
500
1 2 3
1 2 3
1 918
2 964 1813 575
3 1403 971
4 388 590 519
5 420 466 196
6 1329 232 698
7 2620 2066 272
8 484 54
9 596 407 580
10 1036 1051 387
11 334 113 291
12 15
13 26 34 165
14 116 11
15 3 216 761
16 17 1090 24
17 701 128 332
1 613 180
2 323 650 273
3 265 118
4 556 111 131
5 550 138 52
6 471 80 911
7 975 390 109
8 112 31
9 507 1048 555
10 122 1814 1108
11 813 85 153
12 820
13 66 146 41
14 97 100
15 16 49 646
16 11 633 5
17 372 41 373

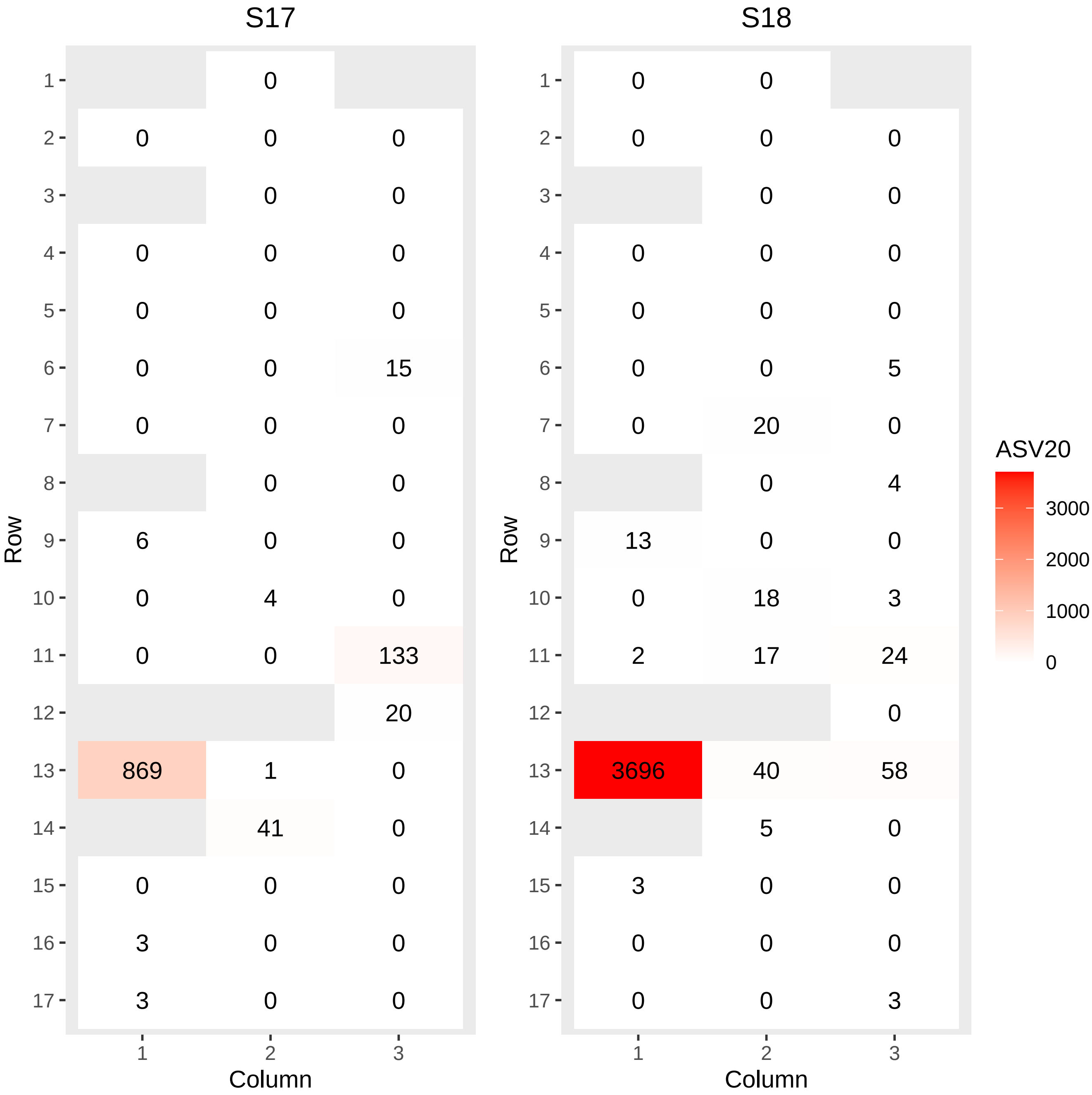

S17
S18
Row
Column
ASV20
3000
2000
1000
0
869
3696

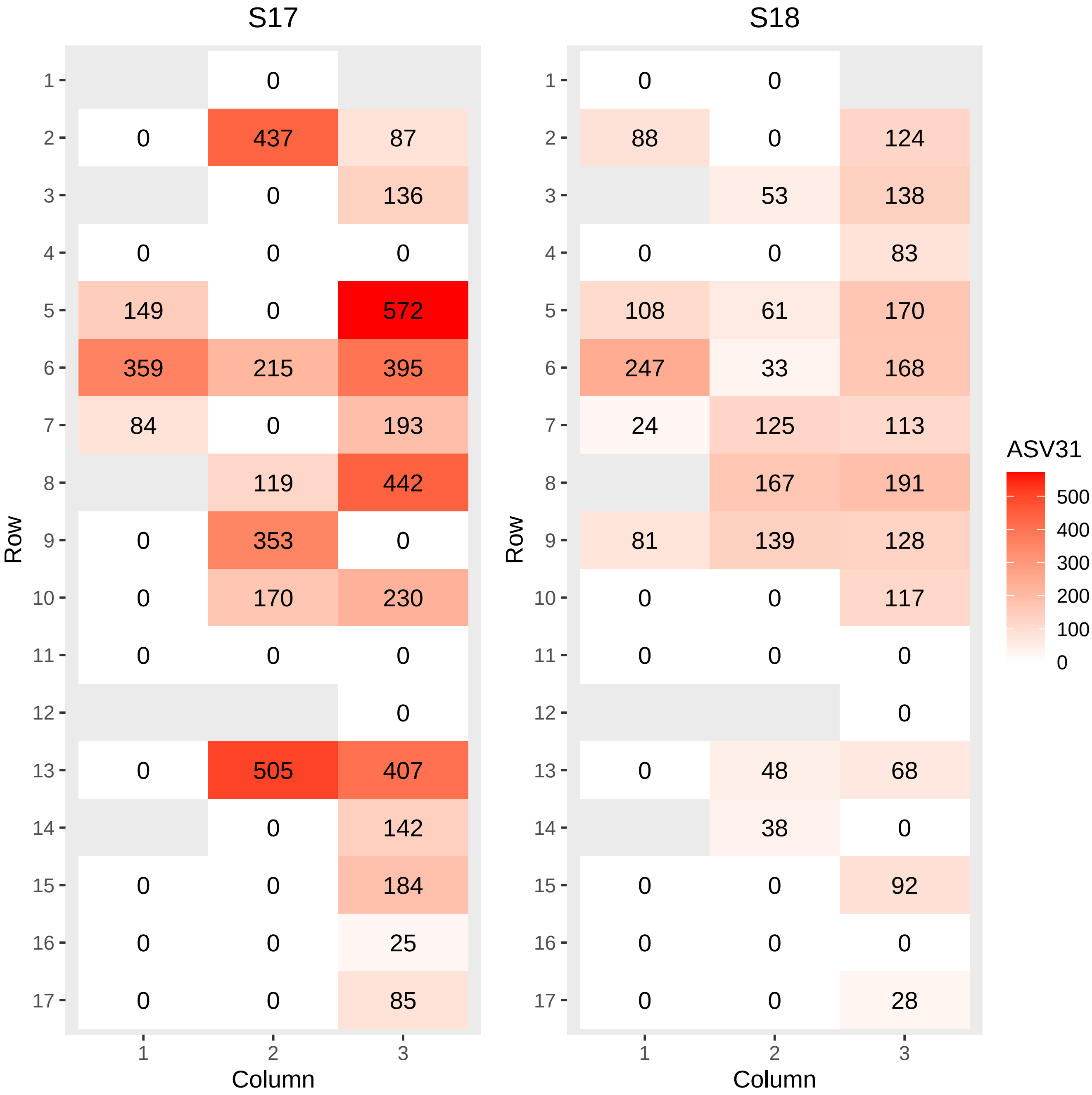

S17
S18
Row
Column
ASV31
500
400
300
200
100
0

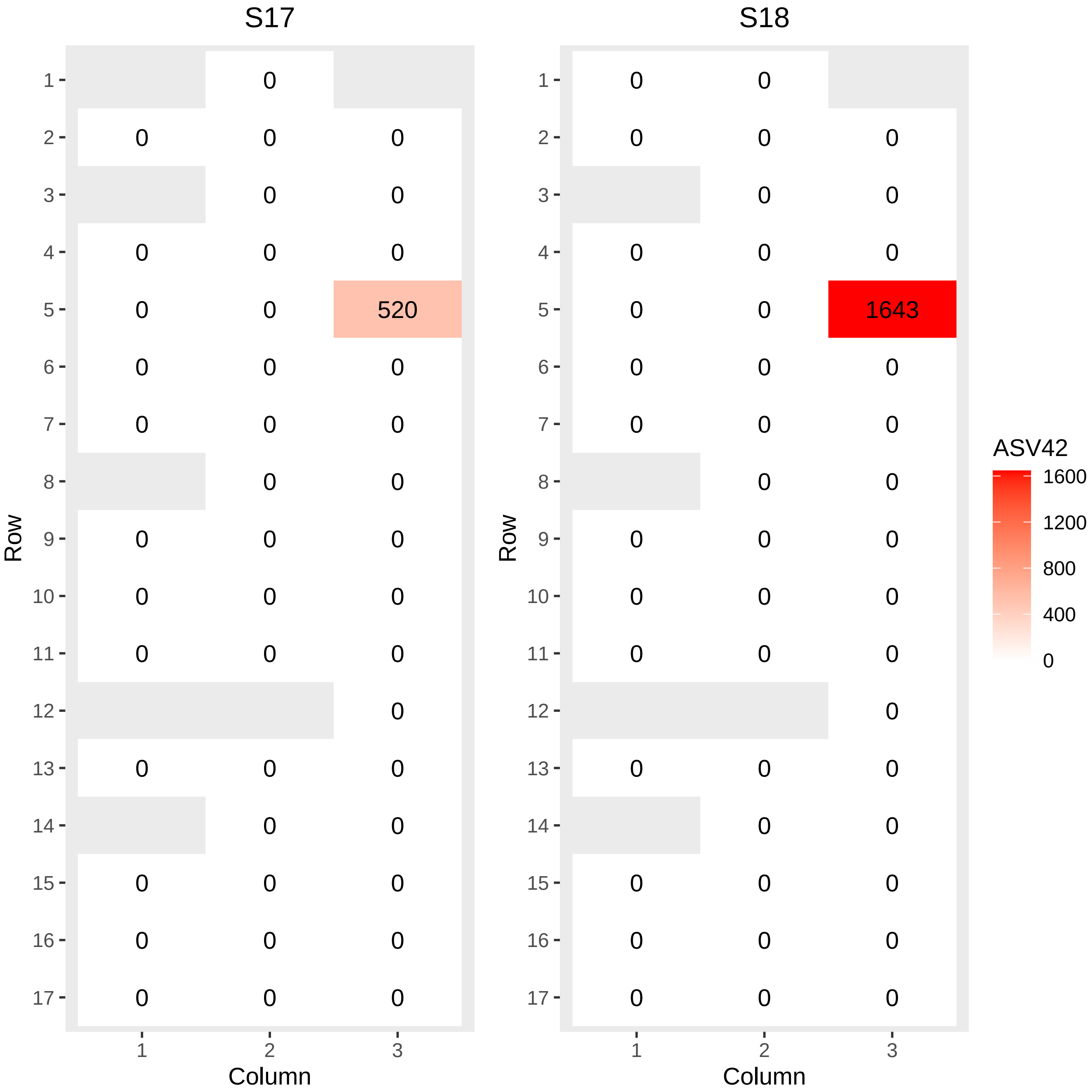
S17
S18
Row
Column
ASV42
1600
1200
800
400
0
520
1643

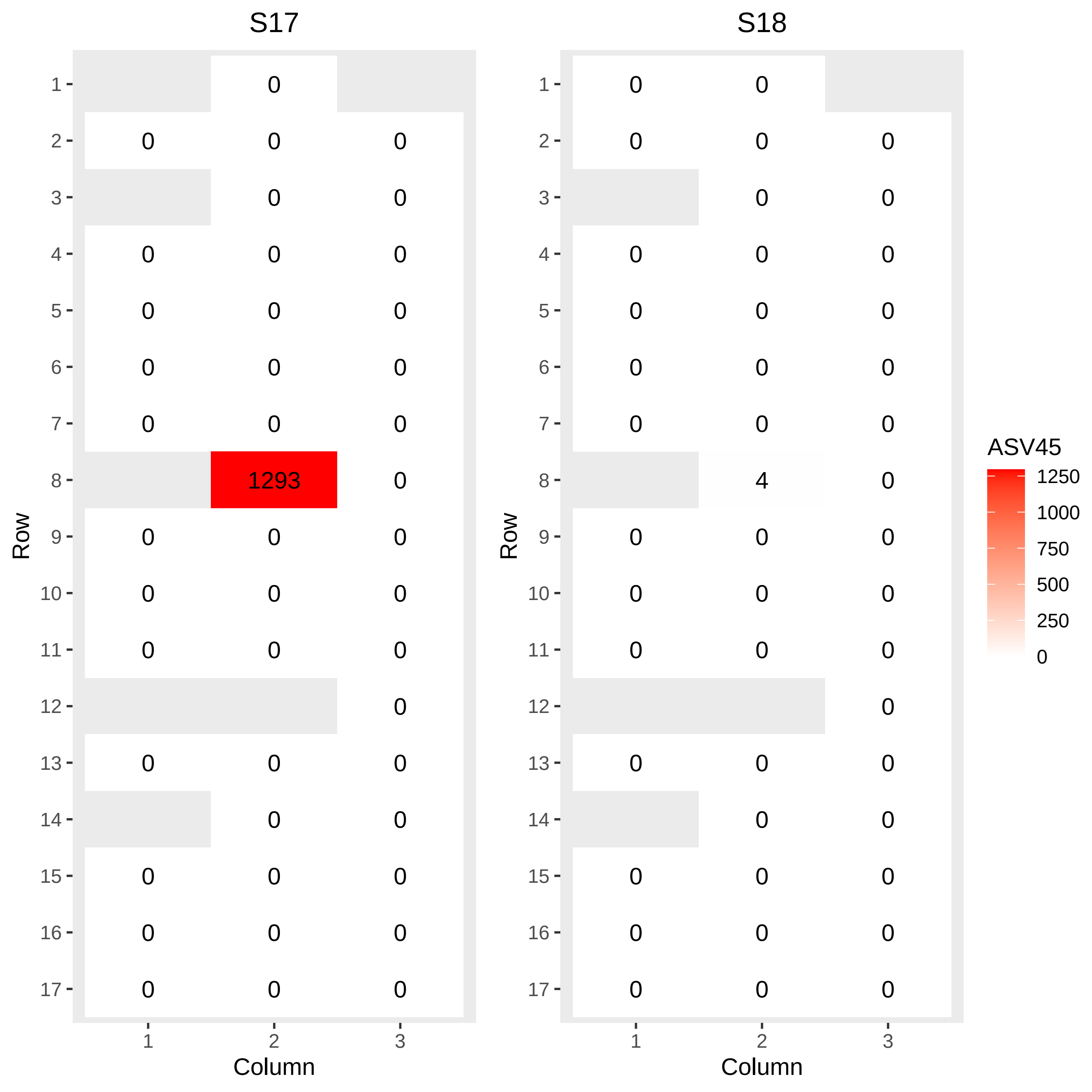
S17
S18
ASV45
1293
4
Row
Column
1250
1000
750
500
250
0

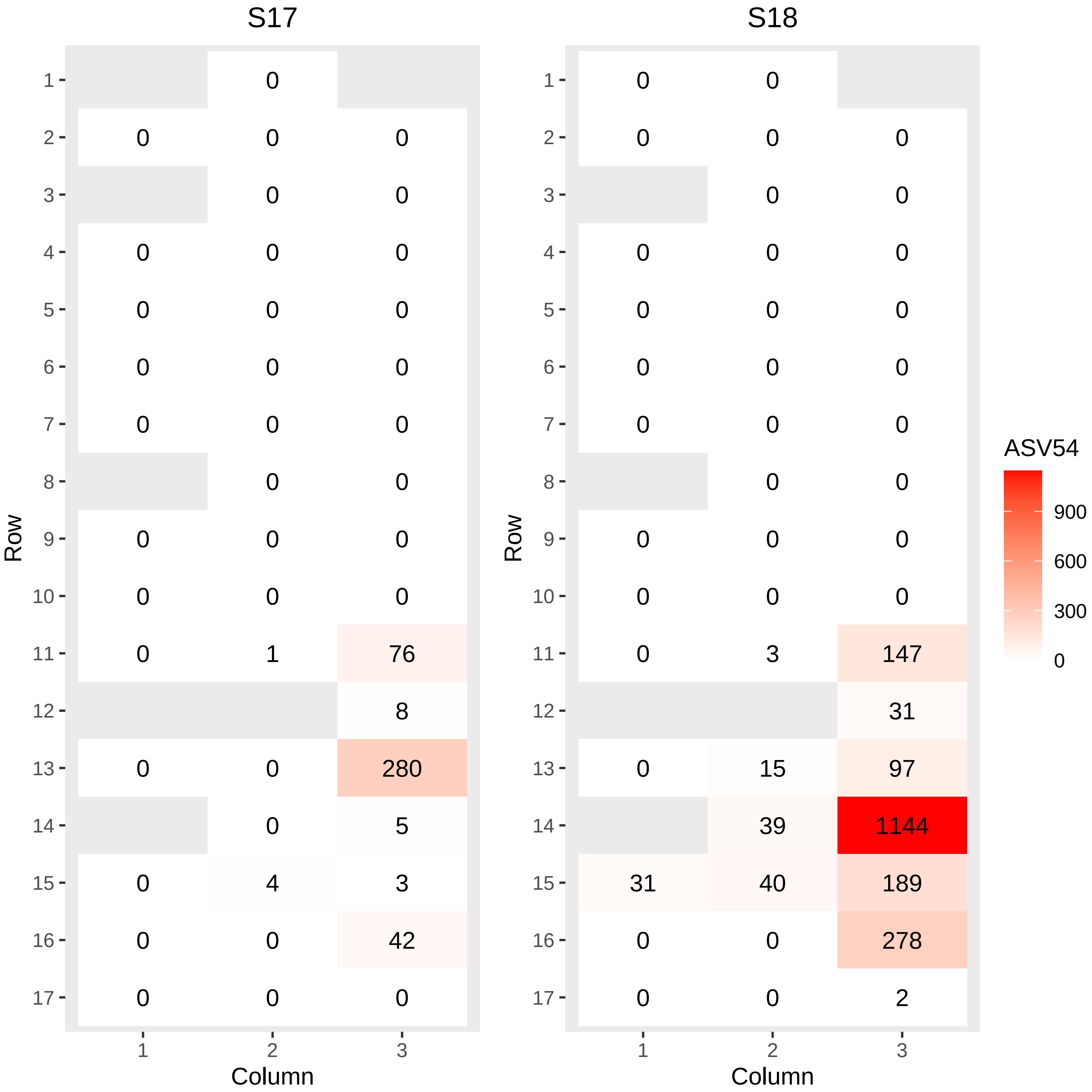
S17
S18
Row
Column
ASV54
900
600
300
0
1
2
3
4
5
6
7
8
9
10
11
12
13
14
15
16
17
0
1
76
8
280
5
4
3
42
147
31
15
97
39
1144
31
40
189
278
2

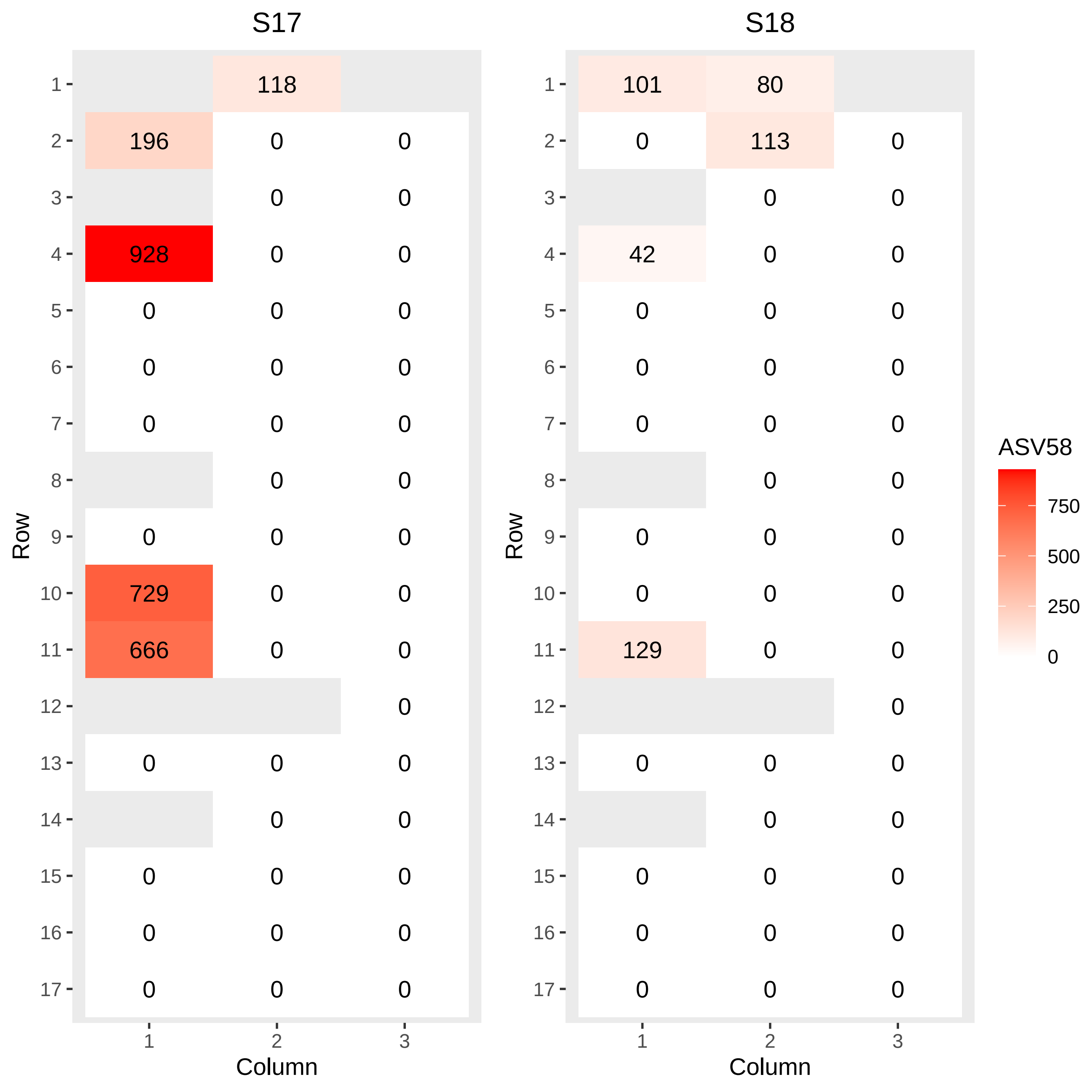

S17
S18
Row
Column
ASV58
750
500
250
0
118
196
928
729
666
101
80
113
42
129

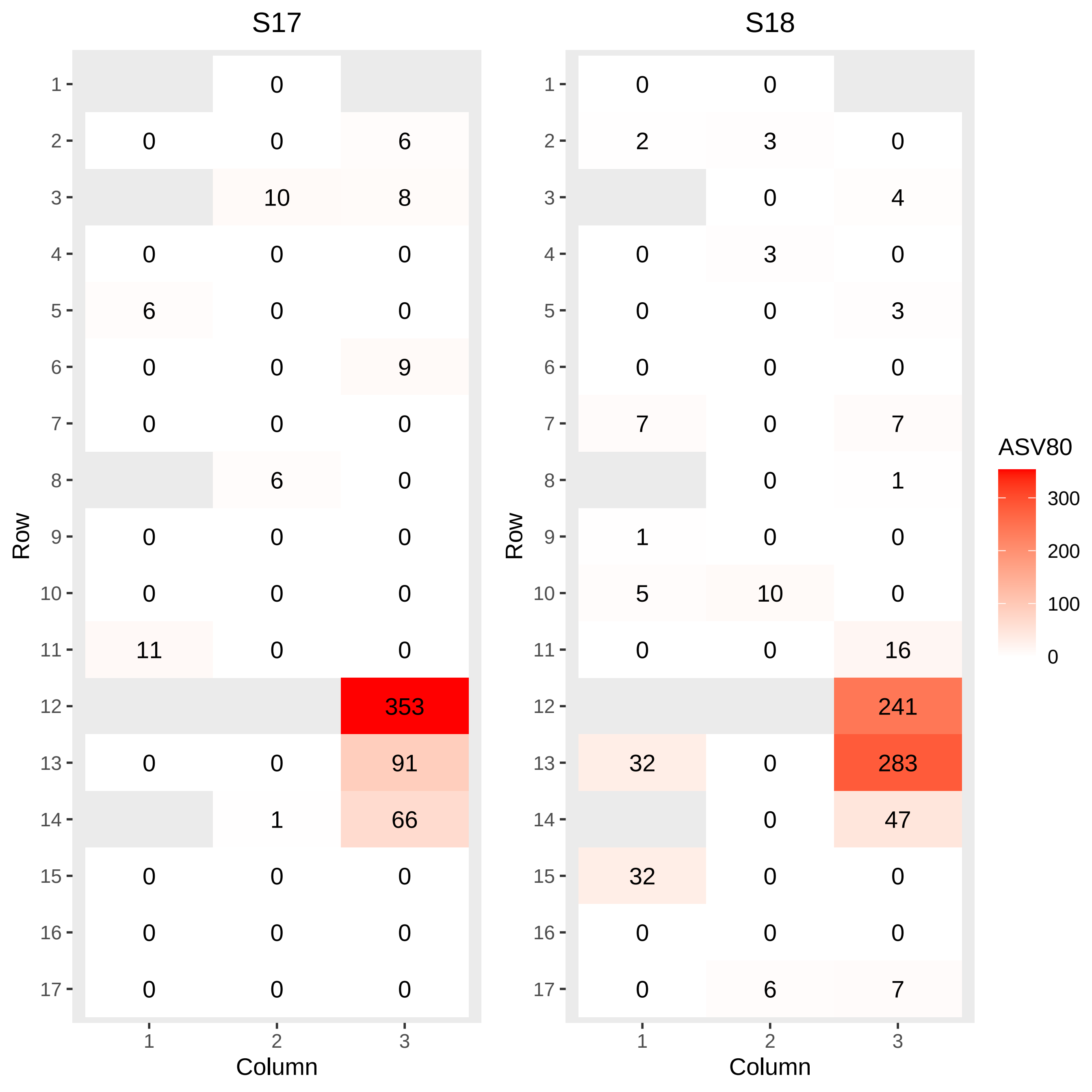

S17
S18
Row
Column
ASV80
300
200
100
0

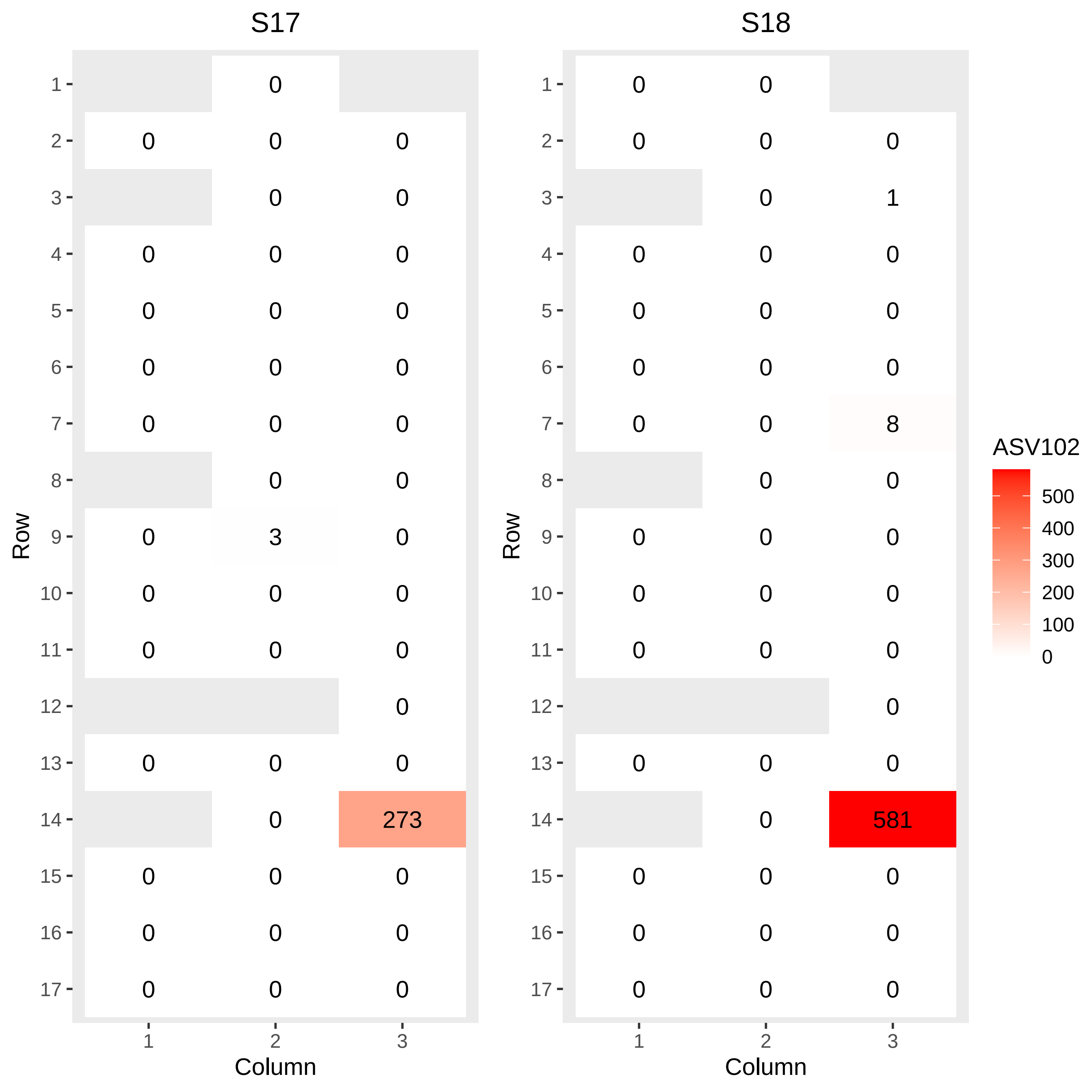

S17
S18
Row
Column
ASV102
500
400
300
200
100
0

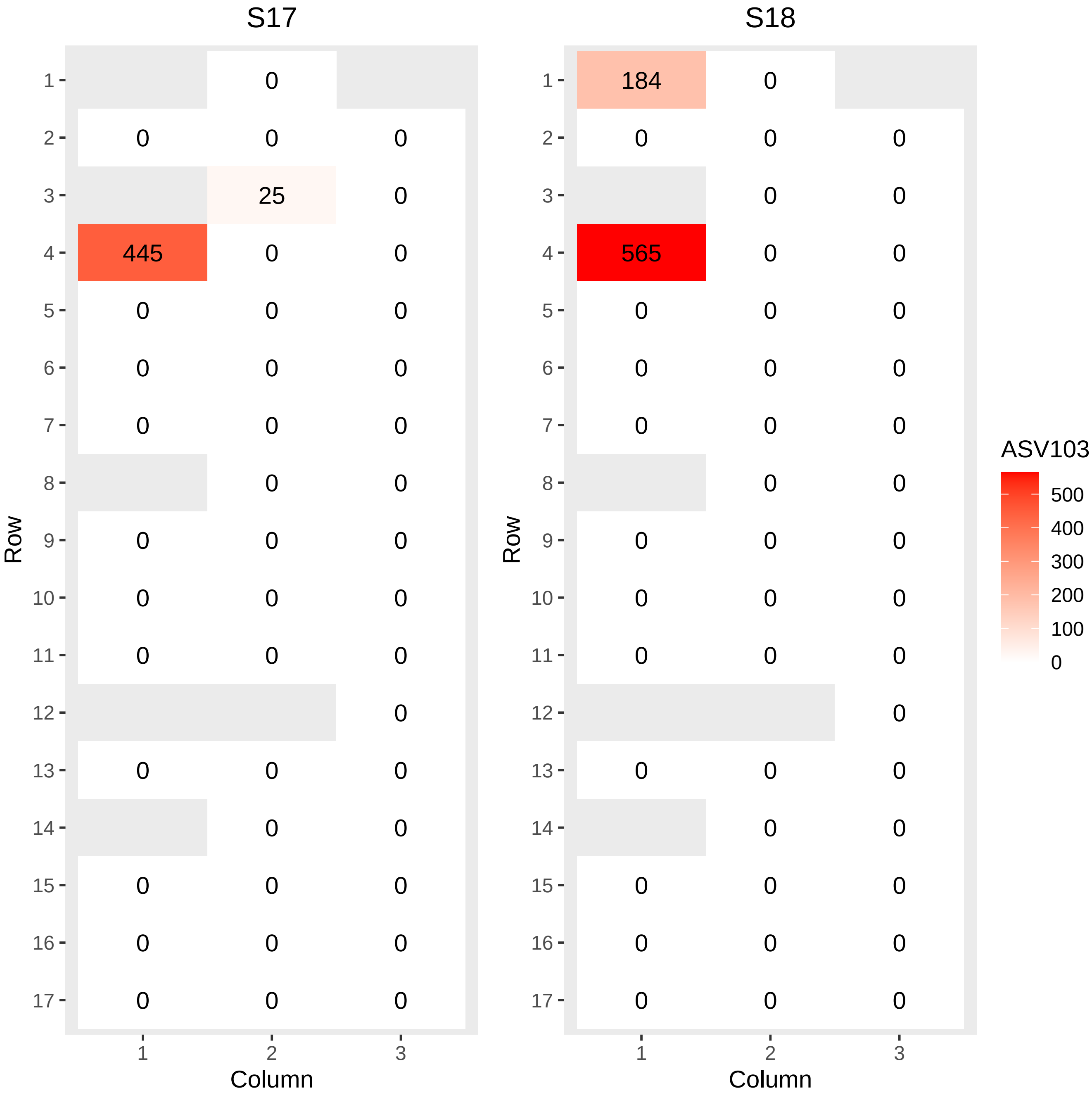
S17
S18
Row
Column
ASV103
500
400
300
200
100
0
184
25
445
565

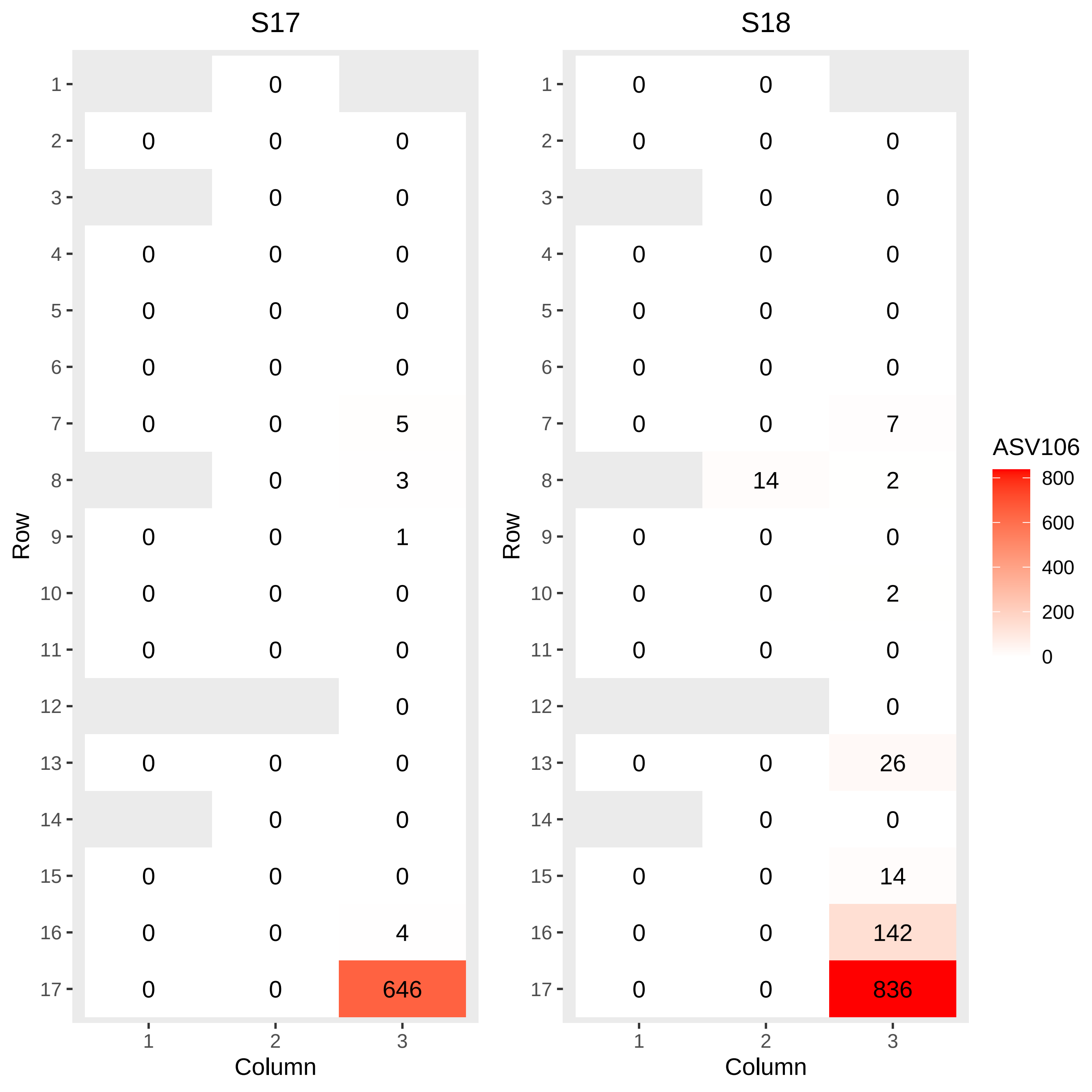

S17
S18
Row
Column
ASV106
800
600
400
200
0
646
836
142
26
14

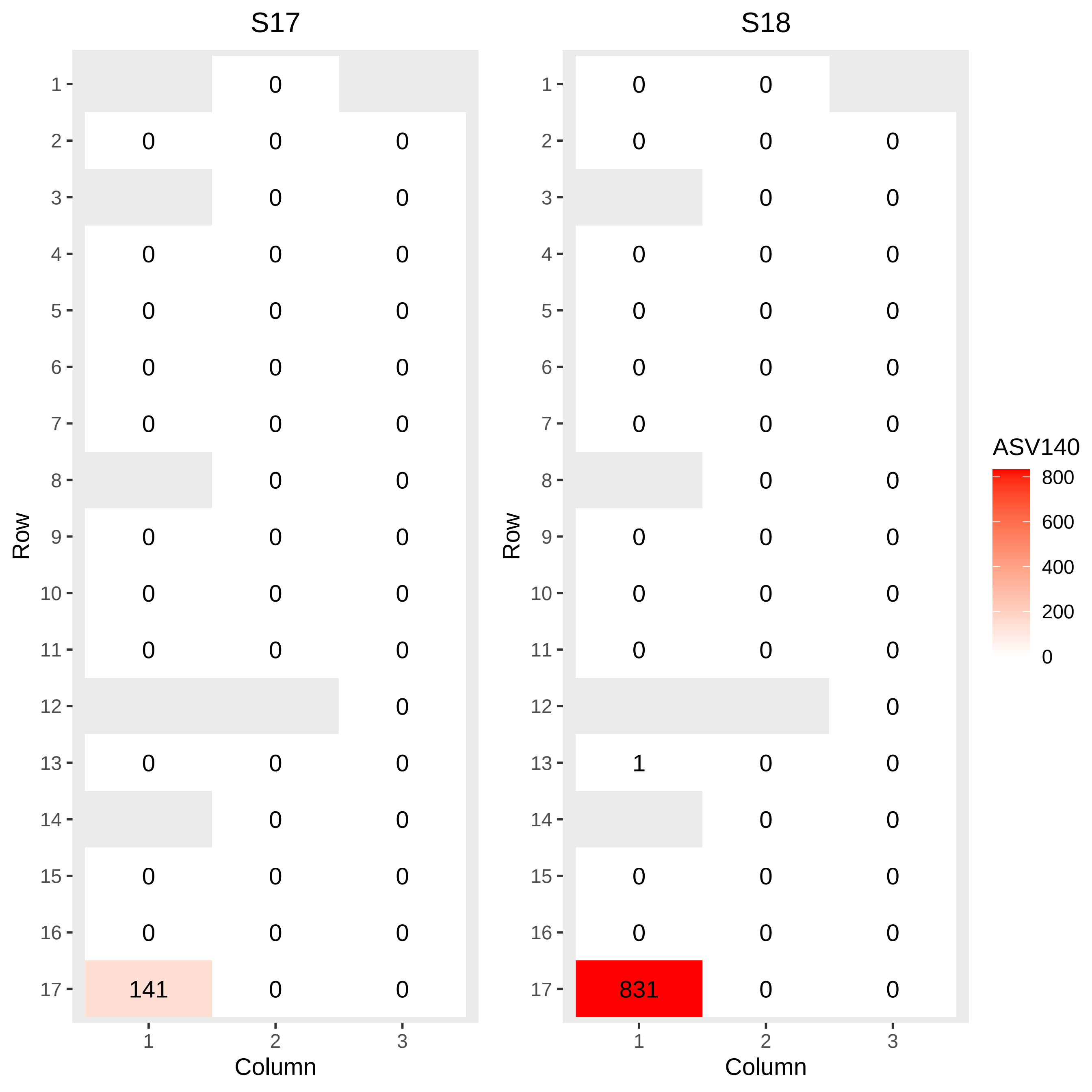
S17
S18
Row
Column
ASV140
800
600
400
200
0
141
831
1

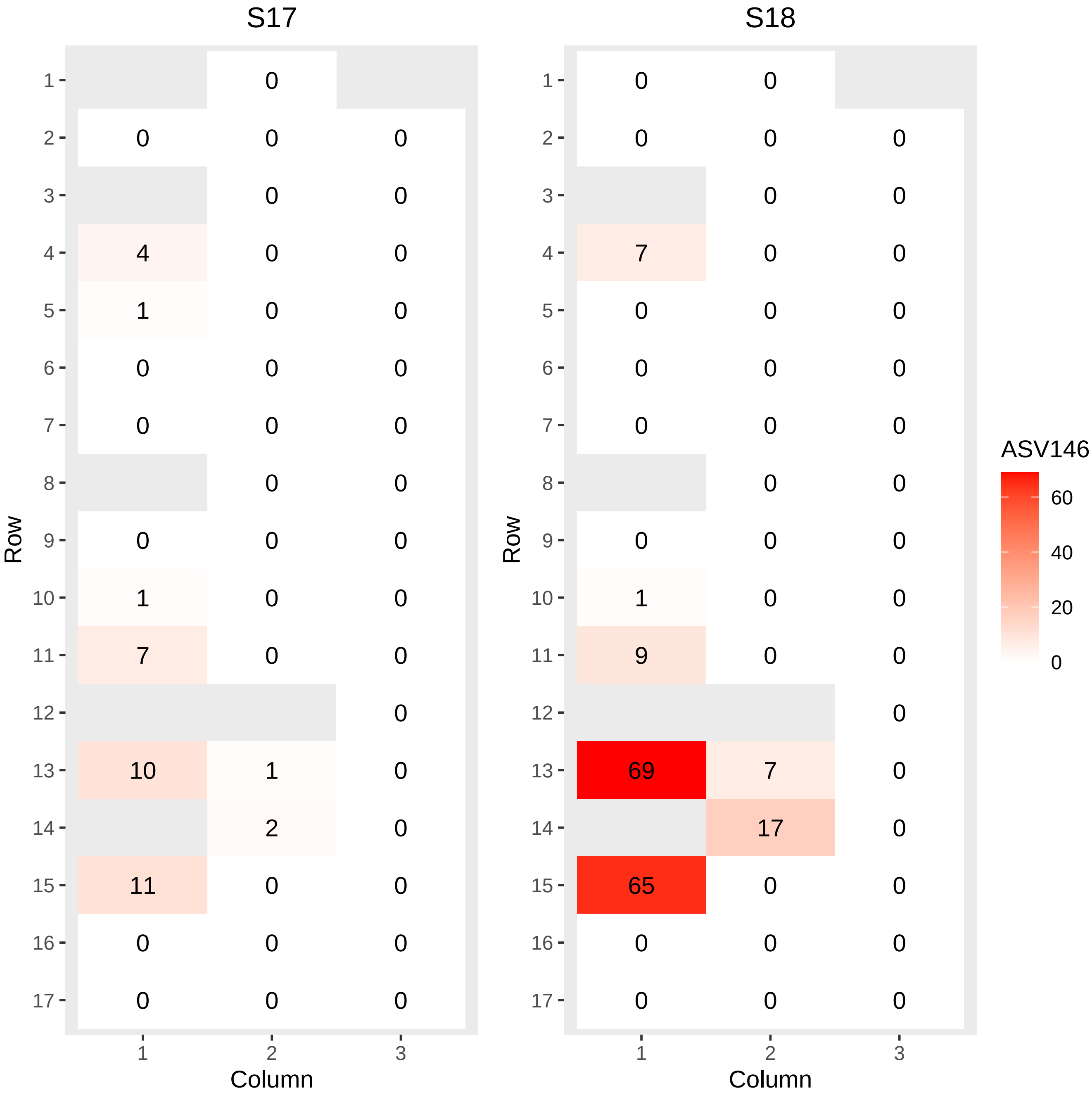
S17
S18
Row
Column
ASV146
60
40
20
0
1
2
3
4
5
6
7
8
9
10
11
12
13
14
15
16
17
69
65
17

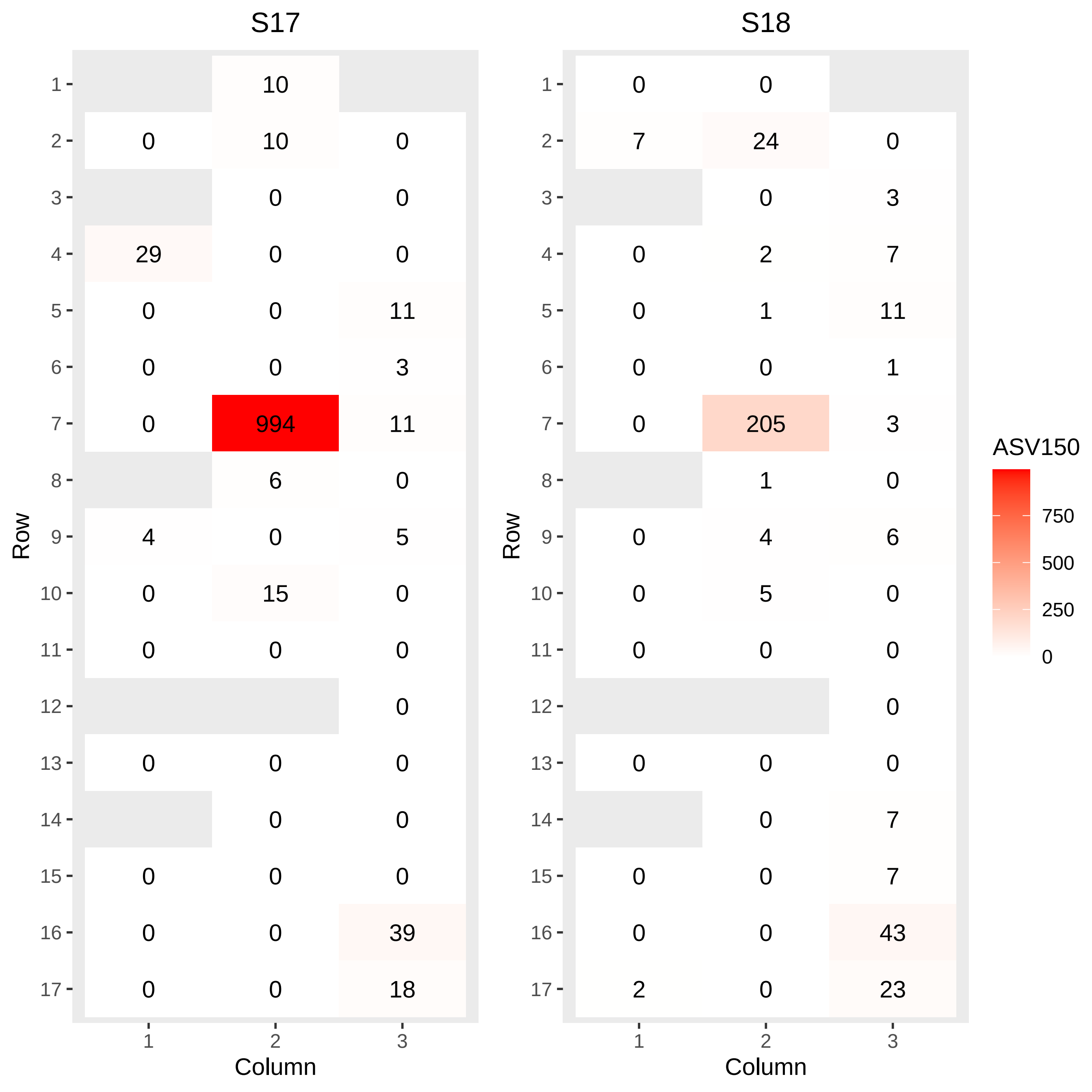
S17
S18
Row
Column
ASV150
750
500
250
0
994
205

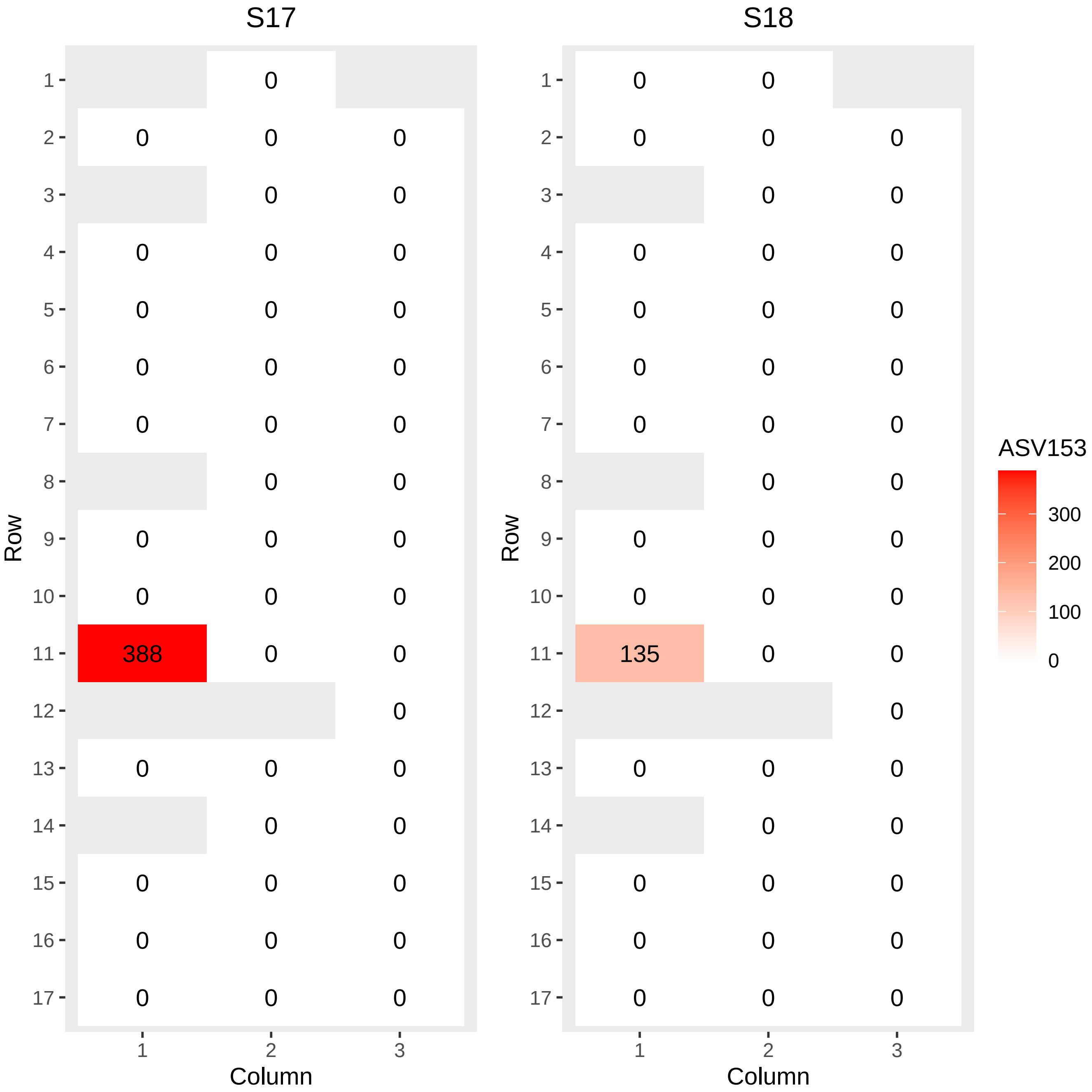
S17
S18
Row
Column
ASV153
388
135
0
100
200
300
